# Longitudinal analysis of lipid changes in the sciatic nerve caused by overexpression of PMP22 in murine models of CMT1A

**DOI:** 10.1016/j.jlr.2026.101018

**Published:** 2026-03-11

**Authors:** Tom P. Hellings, Naima Lamzira-Arichi, Jeroen P. Vreijling, Hailiang Mei, Davy Cats, Tom B. Kuipers, Rico J.E. Derks, Marieke Heijink, Niek Blomberg, Iulia Sidorov, Martin Giera, Frank Baas, Kees Fluiter

**Affiliations:** 1 Department of Clinical Genetics, Leiden University Medical Center, Leiden, The Netherlands; 2 Sequencing Analysis Support Core, Department of Biomedical Data Sciences, Leiden University Medical Center, Leiden, The Netherlands; 3 Center for Proteomics and Metabolomics, Leiden University Medical Center, Leiden, The Netherlands

**Keywords:** lipidomics, genomics, cholesterol/Cell and tissue, plasmalogens and sphingolipids

## Abstract

Charcot-Marie-Tooth type 1A (CMT1A), a prevalent progressive demyelinating peripheral neuropathy is caused by a duplication of the peripheral myelin protein (PMP22) gene. PMP22 is crucial for formation of compact myelin, but the mechanism by which PMP22 overexpression results in CMT1A pathogenesis remains elusive. Emerging evidence points to the role of PMP22 in lipid metabolism as a key modulator of disease progression. Here we show that C3 and C22 mouse models, carrying 5 and 10 additional copies of the human PMP22 gene, have PMP22 dose-dependent lipidomic and transcriptomic alterations. Both models show a decrease in membrane-associated lipids (e.g. phospholipids and sphingolipids) and an increase in neutral lipids (e.g. cholesteryl esters) from three weeks of age. Notably, while cholesteryl ester concentrations are elevated, particularly in C22 mice, total cholesterol levels were significantly reduced, accompanied by the downregulation of key genes involved in cholesterol biosynthesis. Significant decreases were also observed in phospholipids and sphingolipids, including ceramide and sphingomyelin, with a proportional shift towards shorter fatty acid chains in sphingomyelin due to altered ceramide synthase expression. Plasmalogen concentrations decreased with shifts in the proportion of specific plasmalogen species, aligning with impaired synthesis. These lipidomic changes, impacting myelin-associated lipids and fatty acid compositions, underscore their critical role in the dysmyelination observed in CMT1A. Our findings suggest potential avenues for dietary interventions, such as specific fatty acid and plasmalogen supplementation to improve myelination in CMT1A.

## Graphical abstract

**Figure:**
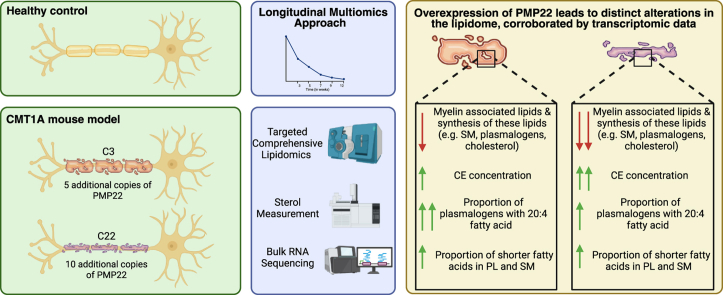

Charcot-Marie-Tooth (CMT) disease is the most common hereditary peripheral neuropathy, affecting approximately 1 in 2500 individuals. Among the various subtypes, CMT1A is the most prevalent (1, 2). Patients with CMT1A have distal muscle atrophy, weakness, and, in severe cases, loss of motor functions and sensory impairments. These symptoms usually manifest within the first two decades of life. Histologically, CMT1A is marked by the loss of peripheral myelinated nerves and frequent segmental demyelination accompanied by the proliferation of Schwann cells. This leads to characteristic “onion bulb” formations around the axons (3, 4, 5). CMT1A is caused by a 1.5 Mb duplication on chromosome 17p11.2, which contains the peripheral myelin protein-22 (*PMP22*) gene (6, 7). PMP22 is a tetraspan membrane glycoprotein highly expressed in myelinating Schwann cells (8, 9, 10). It plays a crucial role in organizing membrane domains rich in cholesterol and sphingolipids, which are essential for compact myelin formation (10).

Although *PMP22* duplication is the established genetic cause of CMT1A, the precise mechanism by which its overexpression leads to demyelination remains elusive. One emerging hypothesis implicates PMP22 in lipid metabolism regulation. PMP22 can bind with cholesterol through specific motifs, cholesterol-recognition amino acid consensus (CRAC) and its inverse CARC (10). Furthermore, PMP22 interacts with two rate-limiting enzymes in cholesterol synthesis, 24-dehydrocholesterol reductase (DHCR24) and 7-dehydrocholesterol reductase (DHCR7) (11). These interactions suggest both direct and indirect roles for PMP22 in lipid regulation. Dysregulation of *PMP22* expression perturbs lipid homeostasis, as *PMP22* knockout mice and fibroblasts from CMT1A patients show increased cholesteryl ester (CE) accumulation, likely due to reduced cholesterol efflux, which is primarily attributed to decreased ATP-binding cassette transporter (ABCA1)-mediated transport in CMT1A cells (12, 13). These accumulations localize near the nucleus within lysosomes and are associated with downregulation of genes involved in triglyceride and cholesterol synthesis (12, 13). Consistent findings in rodent CMT1A models further support this lipid dysregulation. Genes involved in lipid and sterol biosynthesis are significantly downregulated (14, 15). Since cholesterol biosynthesis is a rate-limiting step in myelin production (16), this downregulation could contribute to disease pathology. Moreover, Schwann cell precursors (SCPs) derived from induced pluripotent stem cells (iPSCs) of patients with CMT1A exhibit abnormal lipid handling compared to isogenic controls (15), indicating that lipid dysregulation may begin early in development.

Understanding these alterations is essential for unraveling the underlying disease mechanisms and progression in CMT1A. Previous studies have shown that increasing lipid intake can improve outcomes, enhance axon myelination, and reduce pathological macrophage infiltration and cell proliferation (14, 17). A deeper understanding of PMP22-related lipid metabolism could inform targeted dietary supplementation strategies, including specific fatty acids or lipid species and optimal timing for intervention.

To explore these mechanisms further, we employed two well-characterized mouse models of CMT1A: C22 mice (with 10 additional copies of human *PMP22*) and C3 mice (with 5 additional copies). Both models display low nerve conduction velocities and peripheral nerve demyelination, closely mimicking the human condition (18). The C3 model exhibits milder symptoms than the C22, allowing for analysis of dose-dependent effects of *PMP22* overexpression on lipidomic and transcriptomic profiles, as well as disease severity (18, 19).

Our analysis reveals significant changes in the lipid profile, most notably a reduction in membrane-associated lipids, alterations in the fatty acid composition of the remaining lipid fractions, and an increase in CE and triglycerides (TG). These lipidomic shifts align with changes observed in the transcriptome, offering a plausible explanation for the demyelinating phenotype seen in these animals. Moreover, we observed a dose-dependent effect of the transgene *PMP22* expression, with C22 mice showing more pronounced alterations compared to the C3 mice. Importantly, these findings may extend beyond CMT1A and offer insights into other neurodegenerative diseases. Increasing evidence points to similar lipidome disturbances in disorders such as Alzheimer’s (AD) and Parkinson’s disease (PD), including reductions in sphingomyelin (SM) and phospholipid levels, as well as shifts in comparable fatty acid profiles (20, 21).

## Materials and methods

### Animals

**Fig. 1:**
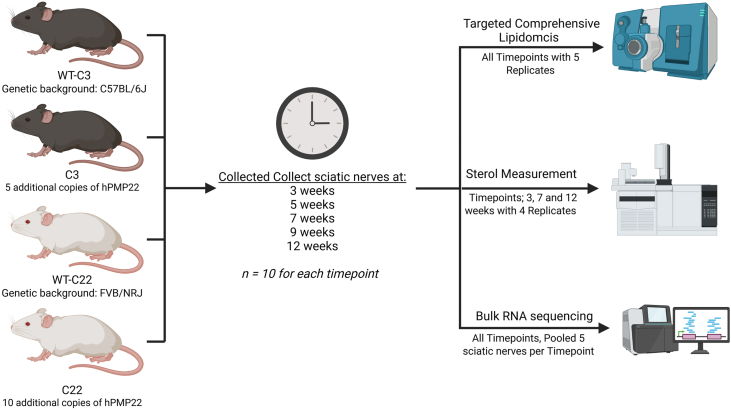
Experimental set-up. A schematic overview of the multi-omics approach. Ten mice from each group, C3, C22, and their respective background-matched controls, were utilized. The C3 mice had a C57BL/6J background, depicted as black mice, and the C22 had a FVB/NRJ background, depicted as white mice. Sciatic nerves were collected at 3, 5, 7, 9, and 12 weeks of age. Targeted lipidomics analyses were conducted with five biological replicates at each time point. Sterol quantification was performed on four replicates per group at 3, 7, and 12 weeks. Bulk RNA sequencing was carried out on a single pooled sample per group and time point. Figure was created in https://BioRender.com.

All animal experiments were done under the supervision of the institutional animal welfare body after ethical approval by the institutional ethical committee and the central (national) commission for animal experiments according to EU directive 2010/63/EU. The C22-*PMP22* mouse model (C22 mice) (19) and its spontaneous partial revertant, the C3-*PMP22* mouse model (C3 mice) (18), were both bred heterozygously using wild-type (WT) males × heterozygous females and kept at Janvier-labs (Le Genest Saint-Isle, France). C22 carries 10 copies of a yeast artificial chromosome (YAC) carrying the human *PMP22* gene; C3 mice have 5 copies. All animals were housed socially with at least three mice per cage. The animal room was maintained at a temperature of 21°C ± 2°C, with a relative humidity between 45% and 65%, and a 12 h–12 h light–dark cycle. All cages were provided with sawdust as bedding material, and food (commercial rodent diet) and drinking water were provided ad libitum. To verify the genotype and copy number of human *PMP22*, we used droplet digital PCR (ddPCR) as reported previously (15). The C3 mice have a C57BL/6J background. While the C22 mice have an FVB/NRJ background, two different control (WT) lines were used. Both sciatic nerves of 10 animals per group at each time point were collected by Janvier-labs and sent to the LUMC (Leiden) on dry ice. A schematic overview of the methods and usage of the sciatic nerves is visualized in Figure 1.

### Analysis of the transcriptome

#### Sample preparation and RNA isolation

For RNA isolation, we pooled the sciatic nerves of 4–5 animals (males and females) per group per time point. The sciatic nerves were homogenized in 1.5 ml Trizol (Life Technologies) and with a Micro-Dismembrator S (Braun Biotech International) for 1 min at 3,000 RPM. After homogenization, we added 300 μl chloroform (VWR) to separate the RNA from the cell debris. The upper phase was mixed with 2-propanol (VWR) and incubated for 10 min. The RNA was pelleted and washed with ethanol, which was air-dried after removal of supernatant. The RNA was purified using the NucleoSpin XS-kit (Macherey-Nagel) and the manufacturer's protocol was followed without the optional filtrate step. All 25 samples were stored at −80 degrees Celsius.

#### RNA sequencing

The RNA sequencing was performed by GenomeScan (Leiden). The samples were treated with DNAse on column, libraries were made using the NEBNext Ultra II Directional RNA Library Prep Kit for Illumina (New England Biolabs). All samples had an RQN score above 7.7. The samples were poly(A) enriched and sequenced on a NovaSeq6000 sequencing, 150 bp Paired-End. Resulting in ∼ 12 GB and around 40 million reads. At least 80% of the datasets should have a Q-score of 30 or higher.

#### RNA mapping and analysis

R-studio version 3.6.3 (2020-02-29) was used for mapping the sequence data and analyzing the raw count data. RNAseq reads were processed using the open-source BIOWDL RNAseq pipeline v5.0.0 developed at the LUMC (https://doi.org/10.5281/zenodo.5109461). This pipeline performs FASTQ preprocessing (including quality control, quality trimming, and adapter clipping), alignment, read quantification, and optionally transcript assembly. FastQC (v0.11.9) was used for checking raw read quality control (QC). Adapter clipping was performed using Cutadapt (v2.10) with the default settings. RNAseq read alignment was performed using STAR (v2.7.5a) on a customized mouse reference genome by adding the transgene YAC locus sequence (human GRCh38 chr17:14929773-15372292) to mouse GRCm39. UMI-based alignment deduplication was performed using umi_tools (v1.1.1). The gene read quantification was performed using HTSeq-count (v0.12.4) with the Ensembl gene annotation version 110, including both all mouse genes and human genes presented in the YAC locus.

Using the gene read count matrix, count per million (CPM) was calculated per sample on all annotated genes. Genes with a log2CPM higher than 1 in at least 25% of all samples are kept for downstream analysis. For the differential gene expression analysis, the dgeAnalysis R-shiny application (https://doi.org/10.5281/zenodo.6979653) was used. EdgeR (v3.28.1) with trimmed mean of M-values (TMM) normalization was used to perform differential gene expression analysis. Benjamini-Hochberg false discovery rate (FDR) was computed to adjust *P*-values obtained for each differentially expressed gene. In addition to the differential gene expression (DGE) analysis, we conducted a time course analysis. For this, the genes were filtered for expression using edgeR's (version 4.2.1) "filterByExpr" function with the default arguments. Counts were then normalized using TMM normalization. Time was represented in the design matrix as a cubic spline produced by the "ns" function of the splines package (version 4.4.1) with 3 degrees of freedom. Per group, *P*-values were calculated for the coefficients of these splines using EdgeR's "glmQLFit" and "glmQLFTest" to identify genes with a time-dependent effect. To identify genes with different time effects between groups, additional coefficients were added to represent the combination of time and group. Again, "glmQLFit" and "glmQLFTest" were used to calculate *P*-values. *P*-values were adjusted using FDR correction. Ingenuity Pathway Analysis (IPA) core analysis (Ingenuity Systems, Qiagen) was used to analyze RNA sequence data and visualize pathways by means of pathway analysis incorporated in IPA (22).

To estimated cell type composition, the bulk RNA sequencing data set was deconvoluted using MuSiC (23), using the single-nuclei data set from Gerber, Pereira (9). To check whether there are differences in the estimated cell types between the groups. There were no major differences in estimated cell type populations between the C3 and the corresponding WT (Supplemental Fig. S1).

### Analysis of the lipidome

#### Targeted comprehensive lipidomics (lipidyzer) and data analysis

Shotgun lipidomics analysis across 17 lipid classes was accomplished by differential mobility spectroscopy coupled with tandem mass spectrometry operated with the shotgun lipidomics assistant software (DMS-SLA platform) (24, 25). The system consisted of a Shimadzu Nexera series LC, coupled to a Sciex QTrap 6500+ equipped with a SelexIon device (Sciex). One sciatic nerve per animal of 5 different mice per group per time point was homogenized and spiked with internal standards (IS), covering 74 deuterated lipid species (UltimateSPLASH™ ONE (Avanti) in combination with oleic acid-d9 (dFA 18:1), C13-dihydroceramide-d7 (d18:0-d7/13:0), C15-glucosyl(β)ceramide-d7 (d18:1-d7/15:0), C15-lactosyl(β)ceramide-d7 (d18:1-d7/15:0), and 15:0-18:1-d7-PA) across 17 different lipid classes (with known concentration for each of them). Lipids were subsequently extracted using a methyl tert-butyl ether (MTBE) (Honeywell Riedel-de Haën) based protocol. Each lipid species was quantified by the SLA software based on its average intensity divided by the average intensity of the most structurally similar IS multiplied by its corresponding concentration. A detailed preparation procedure and the DMS-SLA setting can be found elsewhere (24, 25). Similarly, water blanks and plasma QC were prepared using either LC-MS grade water or human citrate plasma (Sigma P9523) (24, 26). The data was normalized to the protein concentration present in each sample (Supplemental Fig. S2). The data was filtered via http://isoda.online/ (27), lipid species absent in at least 80% of one of the groups at a concentration higher than two times the blank. The total number of lipid species measured was 1071, which was reduced to 866 after filtering. We did not substitute the missing values in the reaming list when depicting our data, as we deem it biologically relevant, especially in the depiction of specific lipid species. Our data had an overrepresentation of TG lipids, while this lipid class is less informative with regard to the demyelinating phenotype observed in CMT1A. Consequently, we removed the TG class from the initial data, the total concentration and TG concentration are shown in Supplemental Fig. S3. The data of C3 and C3-WT including TG can be interactively viewed via; https://cpm-lumc.shinyapps.io/soda-light/?experimentId=NLA_919 and for the C22 and C22-WT via https://cpm-lumc.shinyapps.io/soda-light/?experimentId=NLA_917.

#### Cholesterol measurement

For the analysis of cholesterol and its precursors, nerves were weighed (Supplemental Fig. S4), to normalize the output. Stainless steel beads (0.9–2.0 mm, Next Advance) and reagents were added sequentially to each nerve, after each addition the sample was homogenized in a bullet blender (Next Advance) for 3 min. Sequentially the following reagents were added: 20 μl water (Honeywell Riedel-de Haën), 20 μl ethanol (Supelco, Germany), and 100 μl ethanol containing internal standards (5 μg/ml each of cholestane (Supelco), desmosterol-d6 (Avanti Polar Lipids), and 25-OH-cholesterol-d6 (Avanti Polar Lipids), and 100 μg/ml cholesterol-d7 (Avanti Polar Lipids)). After homogenization, 40 μl ethanol and 20 μl aqueous NaOH solution (10 M) (Sigma) were added. The samples were flushed with nitrogen, vortexed, and incubated at 70°C for 1 h. Thereafter, sterols were extracted using MTBE, and the organic extract was dried using sodium sulfate (Fluka). The extract was subsequently concentrated to dryness under a gentle stream of nitrogen and sterols were silylated using 50 μl derivatization reagent (N-methyl-N-(trimethylsilyl)trifluoroacetamide containing 1% trimethylchlorosilane (ThermoFisher) and N-(trimethylsilyl)imidazole (ThermoScientific) 10:1 (v/v)). Finally, 450 μl MTBE was added. The so-prepared samples were analyzed by GC-MS as described elsewhere (28). Sterols were quantified in single-ion monitoring mode using external calibration lines.

#### Sample preparation and MALDI MS imaging of free cholesterol with MALDI-2-timsTOF flex

A sciatic nerve of C3, WT-C3, C22 and WT-C22 mice at 12 weeks of age were embedded in hydroxypropyl methyl cellulose (HMPC) and poly vinyl pyrrolidone (PVP) (29). Embedded tissue was cryo-sectioned at 10 μm thickness (using a CM3050 S, Leica Biosystems) and mounted onto one indium tin oxide (ITO) slide (VisionTek Systems Ltd). The slide was vacuum freeze-dried for 15 min, after which 18 layers of 500 μg/ml cholesterol-D7 (Avanti) in absolute ethanol were sprayed on the tissues using the SunCollect sprayer (20 μl/min flow rate, 30 mm Z position, 2 mm line distance, x and y linear velocity = medium 1). After which, 2,5-dihydroxybenzoic acid (DHB) matrix in pure acetone (50 mg/2 ml) was sublimated using HTX sublimator (HTX Technologies, LLC). The MALDI-2-timsTOF flex instrument acquired positive ion mode spectra with a mass range of m/z 200–500, using 5 μm spatial resolution (22% laser power and 15 shots per pixel). MALDI-2 mode was used with a trigger delay of 5.0 μs and an acquisition speed of 1 kHz. Red phosphorus was used for calibration, after which data acquisition was performed using TIMSontrol and flexImaging 7.3. The Mass Spectrometry Imaging data were loaded into SCiLS Lab (Version 2026a Pro, Bruker Daltonics). The data were normalized against the peak area of the cholesterol-D7 (m/z 376.39) internal standard. MS images were generated for the cholesterol ion (m/z 369.35).

#### Statistical analysis

All data was tested for normality of the residuals with a Shapiro-Wilk test. Two-way ANOVA followed by a Tukey post-hoc test was performed on the lipidomics data using GraphPad Prism (version 10.2.3, GraphPad Software, Boston, Massachusetts USA, www.graphpad.com). To visualize the proportional shifts in CMT1A, directional analysis of the lipidome was conducted in BioPan (30). LipidLynxX was used to change the nomenclature of the lipid species for BioPan usage (31). The annotation level was reduced by summing all the fatty acid chains of the lipid species to make it one. This resulted in 288 lipid species used in the analysis, and 89 being unprocessed. The data of each mouse model was averaged to see general trends in lipidome changes. The z-score was calculated by computing a weight value to determine the direction of the synthesis pathway. This is done by calculating a ratio of product over reactant for the control and the condition of interest. For each weighted edge, a *t* test is conducted; by assuming normality, the *P*-value can be converted to a z-score (30).

## Results

### Reduced cholesterol synthesis and sterol concentration in the sciatic nerve of CMT1A mouse models

**Fig. 2:**
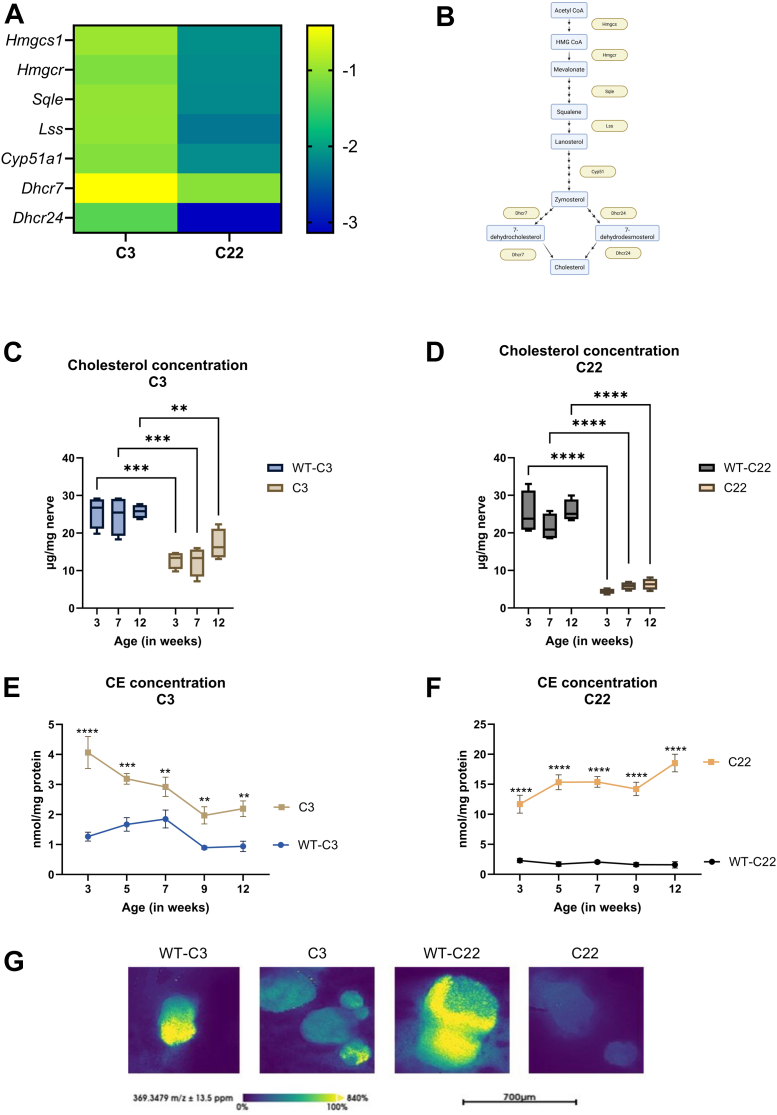
Cholesterol and cholesteryl ester over time in the sciatic nerve of two different CMT1A mice models with downregulated expression of rate limiting enzymes. A: A heatmap of the log2(Fold change) of differentially expressed genes in C3 and the C22 compared to the corresponding controls of rate-limiting genes involved in the de novo synthesis of cholesterol. B: Visualized is a simplified synthesis pathway of cholesterol from Acetyl CoA towards cholesterol with important genes within this synthesis pathway and cholesterol precursors. The figure was created in https://BioRender.com. C & D: The cholesterol concentration in the sciatic nerve normalized for the weight of the sciatic nerve (in mg) of C3 and C22 compared to age-matched controls. E & F) Cholesteryl ester (CE) concentration normalized to the protein concentration (in mg) in C3 and C22 compared to age matched controls. Data is visualized by means of a boxplot ± SEM and bar graph ± SEM, analysis was performed using a two-way ANOVA with a Tukey post hoc analysis (∗*P* < 0.05, ∗∗*P* < 0.002, ∗∗∗*P* < 0.0002, ∗∗∗∗*P* < 0.0001). G) MALDI MS image of free cholesterol was taken of the sciatic nerve of 12-week-old C3 and C22 and corresponding controls. A clear reduction of free cholesterol is observed in C3, with an even more pronounced effect in C22. MGCS(1), 3-Hydroxy-3-Methylglutaryl-CoA Synthase 1; HMGCR, 3-hydroxy-3-methylglutaryl CoA reductase; SQLE, Squalene Epoxidase; LSS, Lanosterol synthase; CYP51, Lanosterol 14α-demethylase; DHCR7, 7-Dehydrocholesterol Reductase; DHCR24, 24-Dehydrocholesterol Reductase.

Cholesterol is a major component of myelin and vital for the formation of compact myelin. Consequently, a downregulation of cholesterol synthesis and concentration can severely impact the CMT1A pathology. We show severe downregulation in cholesterol biosynthesis and the super-pathway of cholesterol biosynthesis in C3 and C22 (Table 1). A consistent downregulation of individual genes involved in the cholesterol synthesis pathway is observed in C3 and C22 compared to their corresponding WT counterparts (Fig. 2A, B), which is in line with previous work (15). In the control mice, expression levels of genes involved in the cholesterol synthesis pathway are negatively correlated with age, showing a downward trend from 3 weeks of age. While in the sciatic nerve of the C22 and C3 mice, the expression of rate-limiting enzymes (*Sqle* and *Dhcr24* in the C3 and *Hmgcr*, *Lss*, and *Dhcr24* in the C22) was unaffected by age (Supplemental Fig. S5A, B).

**Table 1:** Differential expression of lipid synthesis pathways in the sciatic nerves of CMT1A mice models compared to controls

Lipid Metabolism Pahtways	C3 versus WT-C3		C22 versus WT-C22	
	-log(*P*-value)	Z-score	-log(*P*-value)	Z-score
Cholesterol biosynthesis	11.5	−3.77	12.9	−4.26
Superpathway of Cholesterol Biosynthesis	10.4	−4.12	11.3	−4.58
Glycerophospholipid biosynthe sis	2.25	−2.29	2.78	−2.74
Sphingolipid metabolism	6.89	−1.07	7.23	−2.07
Oleate Biosynthesis II	2.51	−2.24	3.3	−2.65

Bulk RNA sequencing data of the sciatic nerve of C3 and C22 and corresponding controls are analyzed with ingenuity pathway analysis (IPA). Showing significant downregulation in multiple pathways: Cholesterol biosynthesis, Super pathway of Cholesterol Biosynthesis, Glycerophospholipid biosynthesis, Sphingolipid metabolism, Oleate Biosynthesis II in C3 and C22 compared to the corresponding control.

To further substantiate our findings, we show significantly reduced cholesterol concentrations in the sciatic nerves of C3 and C22 relative to wildtype counterparts across all time points (Fig. 2C, D). The CE concentration was significantly elevated in both C3 and C22 mice across all time points (Fig. 2E, F). We do observe a decrease in CE concentration over time in the C3 animals, while in the C22 the CE accumulation increases over time (Fig. 2E, F). MALDI MS images of free cholesterol (m/z 369.35) on coupes of the sciatic nerves, showing a reduction of free cholesterol in both C3 and C22 mice compared to corresponding WT (Fig. 2G). Notably, these differences are amplified by a higher *PMP22* copy number. The concentrations of cholesterol precursors; lathosterol and cholestanol, were lower in C3 compared to age-matched WT (Supplemental Fig. S6A). However, desmosterol concentration did not differ between C3 and WT (Supplemental Fig. S6A). In C22 mice, zymostenol, desmosterol and lathosterol concentrations were below the detection limit. Cholestanol was lower compared in the C22 at 7 and 12 weeks of age compared to age-matched WT (Supplemental Fig. S6B).

### PMP22 dose-dependent decrease in myelin-associated lipids and increase in neutral lipids

**Fig. 3:**
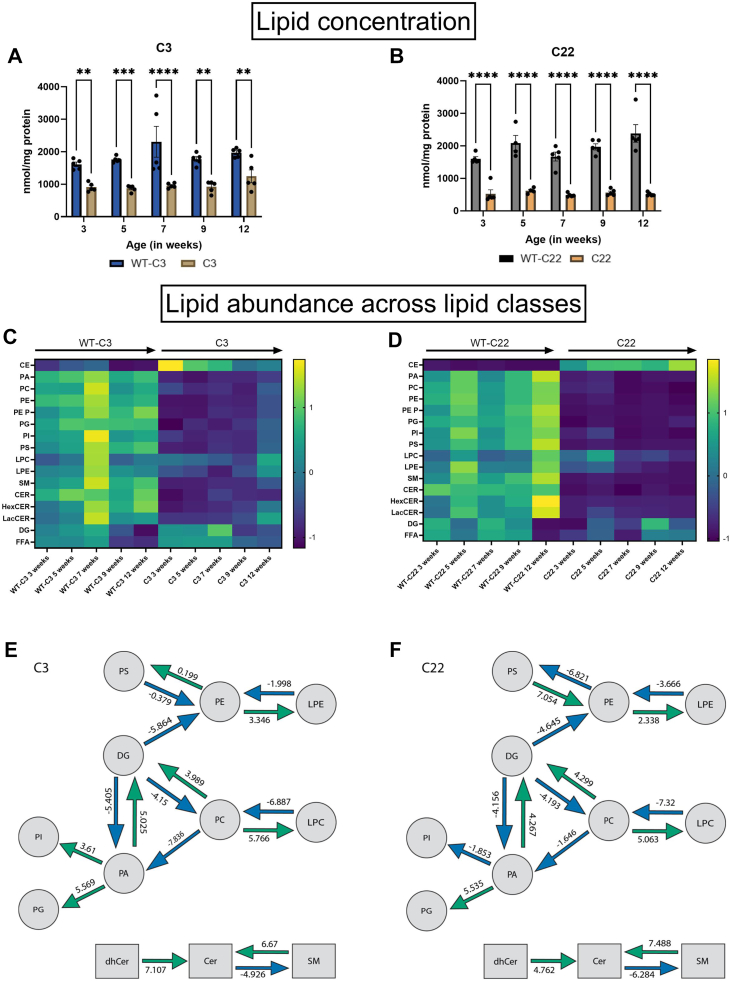
Altered lipid concentration and synthesis pathways based on the lipidome of two different CMT1A models compared to controls. A & B: Lipid concentration of the sciatic nerve of the C3 and the C22 at 5 time points (3, 5, 7, 9 and 12 weeks of age) compared to matched controls. Data is visualized as bar graph ± SEM, analysis was performed using a two-way ANOVA with a Tukey post hoc analysis (∗∗*P* < 0.002, ∗∗∗*P* < 0.0002, ∗∗∗∗*P* < 0.0001). C & D: Heatmap of the z-scores of the lipid class concentration corrected for protein concentration for the C3 and C22, and the corresponding control. E & F: BioPan analysis showed changes in both the C3 and C22 compared to controls. The z-scores were calculated by computing a weight value to determine the direction of the synthesis pathway. For each weighted edge a *t* test is conducted; by assuming normality, the *P*-value can be converted to a z-score. CE, cholesteryl esters; CER, Cerides; dhCER, dihydroceramide; HexCER, Hexosylceramid and LacCER, Lactosylceramid; LPC, Lyso-phosphatidylcholine; LPE, Lyso-phosphatidyl-ethanolamines; PA, phosphatidate; PC, phosphatidylcholine; PE, phosphatidylethanolamine; PE p-, plasmenylethalomines; PG, Phosphatidylglycerols; PI, Phosphatidyl-inositol; PS, Phosphatidyl-serine; SM, Sphingomyeline.

The lipid concentration measured with the Lipidyzer, excluding TG, is consistently lower in both the C3 and C22 relative to their wildtype counterparts (Fig. 3A, B). The overall decrease in lipids is due to a lower concentration of myelin-associated lipids, phospholipids and sphingolipids (Fig. 3C, D). Diglycerides (DG) and free fatty acids (FFA) are less affected in C3 and C22 (Fig. 3C, D). In contrast, the CE concentration in the C3 and C22, suggesting a shift in cholesterol metabolism towards lipid storage rather than remyelination. All observed changes are more pronounced in C22 compared to C3 mice. Notably, myelin-associated lipids in the C3 are lower at 3 weeks of age, when myelination still occurs, compared to the sciatic nerves of the C3 at 12 weeks of age (Fig. 3C, D).

To gain insight into the proportional shifts of the lipidome, we conducted directional analysis using BioPan. We observed significant shifts in activated and inactivated lipid metabolism pathways for the C3 and C22 compared to the corresponding WT (Fig. 3E, F). In both the C3 and the C22 the conversion from SM to ceramide (CER) was in the top 5 most activated pathways (Fig. 3E, F). This activation was accompanied by a shift from dihydroceramide (dhCER) to CER, while the conversion from CER towards SM was suppressed in the C3 and C22 compared to the corresponding WT (Fig. 3E, F). The conversion from phosphatidylcholine (PC) to lysophosphatidylcholine (LPC) and from phosphatidylethanolamine (PE) to lysophosphatidylethanolamine (LPE) was increased in both C3 and C22 mice, while the conversion from LPC to PC and from LPE and PE was decreased in both the C3 and the C22 compared to the corresponding wildtype (Fig. 3E, F). Showing that there is a proportional shift towards lysophospholipids as a result of *PMP22* overexpression.

### Reduced phospholipid concentration and shift toward phosphatidylcholine in the CMT1A mouse models

**Fig. 4:**
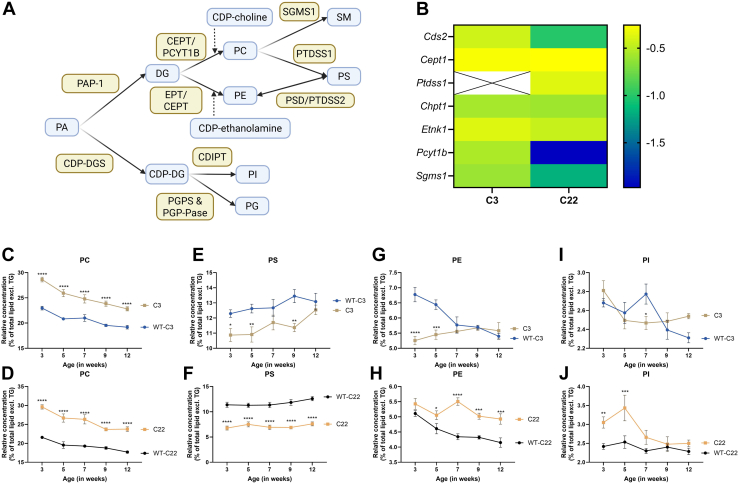
Concentration of phospholipids measured in the sciatic nerve of two different CMT1A mouse models. A: Visualized is a simplified synthesis pathway of phospholipids from phosphatidic acid (PA) towards multiple phospholipids. Figure was created in https://BioRender.com. B: A heatmap of the log2(Fold change) of differentially expressed genes in C3 and the C22 compared to the corresponding controls, showing reduced expression of genes involved in phospholipid synthesis in the C3 and C22 mice compared to the corresponding controls. C-J: Concentration per mg protein divided by the lipidome (excluding TG) of multiple phospholipid species over time in the sciatic nerve of the CMT1A mice models C3, C22 and the corresponding control. Data is visualized as the mean ± SEM, analysis was performed using a two-way ANOVA with a Tukey post hoc analysis (∗*P* < 0.05, ∗∗*P* < 0.002, ∗∗∗*P* < 0.0002, ∗∗∗∗*P* < 0.0001). CDP-DG, CDP-diacylglycerol; CDP-DGS, CDP-DG synthase; CDS1/2, CDP-diacylglycerol synthase half; CEPT, 1,2-diacylglycerol cholinephosphotransferase; CHPT1, Choline Phosphotransferase 1; DG, diacylglycerol; EPT, ethanolaminephosphotransferase; ETNK1, Ethanolamine Kinase 1; PAP-1, phosphatase-1; PC, phosphatidylcholines; PCYTB1B, Phosphate Cytidylyltransferase 1B Choline; PE, phosphatidylethanolamine; PGP-Pase, PGP phosphatase; PGPS, PGP synthase; PI, phosphatidylinositol; PS, phosphatidylserine; PTDSS1, Phosphatidylserine Synthase,1; PTSS1, Phosphatidylserine synthase 1; SGMS1, SM synthase-1.

Given the substantial alterations observed in phospholipid profiles in the C3 and C22, we wanted to further investigate this by looking at phospholipid synthesis and proportional shift within the lipidome. A clear decrease in the sum of phospholipid species (normalized to protein concentration) in the C3 and C22 compared to the corresponding WT was observed (Supplementary Fig. S7A, B). This overall reduction is supported by changes in the transcriptome, showing severe down regulation of the Glycerophospholipid biosynthesis pathway in IPA, a synthesis pathway of numerous phospholipids and plasmalogens (Table 1), this is supported by the reduced gene expression in the phospholipid synthesis pathway as shown in Figure 4A, B. Among the various phospholipid classes, PC is the most abundant. PC constitutes a larger proportion of the lipidome in both the C3 and C22 compared to the controls (Fig. 4C, D). Notably, the proportional increase occurs despite the impaired synthesis of PC in the C3 and C22. The expression of three key enzymes: choline/ethanolamine phosphotransferase (*Cept1*), the paralog choline phosphotransferase 1 (*Chpt1*), and phosphate cytidylyltransferase 1B (*Pcyt1b*), are downregulated compared to WT controls. However, PC degradation is also reduced in the C22, as the expression of phosphatidylserine synthase 1 (*Ptdss1*) and sphingomyelin synthase 1 is down in both the C3 and C22 (Fig. 4B). Moreover, the expression of an enzyme catalyzing the hydrolysis of PC and PE phospholipase A2 (*Pla2*) is decreased in both C3 and C22 compared to WT, with only *Plag6* expression being upregulated in the C3 nerves (Supplementary Fig. S8). This suggests that the proportional shift towards PC is a result of hampered degradation of lipids. In addition, the proportional concentration of LPC was elevated in the C3 nerves compared to the corresponding WT. For the C22 the relative concentration was higher except at 12 weeks of age (Supplementary Fig. S9A, B). The downregulation in *Pla2* expression and increase in LPC concentration is accompanied with changes in LPC and PC lipid species. In which palmitoleic acid (16:1) and oleic acid (18:1) are less abundant in both the C3 and C22 compared to the corresponding controls (Supplementary Fig. S10A–D). In the PC lipid class the proportion shifted towards poly unsaturated fatty acids, this shift is more pronounced in C22 mice compared to C3 mice (Supplementary Fig. S10A, B). Within the LPC lipid class the reduction of 16:1 and 18:1 are accompanied by a relative increase of palmitic acid (16:0) and stearic acid (18:0) (Supplementary Fig. S10C, D), which is in line with the reduce *Pla2g5* expression (Supplementary Fig. S8). However, the increase in LPC with poly unsaturated fatty acids was only consistently present in the C22. Contrary, we observed elevated expression of lysophosphatidylcholine acyltransferase 2 (*Lpcat2*), an enzyme catalyzing the reacylation of LPC to PC (Supplementary Fig. S8).

Phosphatidylserine (PS), the second most abundant phospholipid in the sciatic nerve, has a lower relative concentration in the C3 mice at 3, 5 and 9 weeks (Fig. 4E), while it is lower for all timepoints in the C22 compared to WT (Fig. 4F). The genes *Psd1* and *Ptdss2*, encoding for key enzymes converting PS to PE, are not differentially expressed. While, as previously stated, the expression of *Ptdss1* (encoding for PSS1, a key enzyme for the conversion of PC to PS) is lower in C22 mice (Fig. 4B), potentially leading to this overall shift. Within the PS, there is a reduced proportion of 18:1 fatty acid in the C3 and the C22 compared to the controls, which is accompanied by a proportional increase in 18:0 fatty acids (Supplementary Fig. S11A, B).

PE is synthesized from diacylglycerol by ethanolamine phosphotransferase (EPT) and CEPT1, of which *Cept1* is significantly lower expressed in the C3 and C22 (Fig. 4B). In line, the proportion of PE is lower at 3 and 5 weeks of age in the C3. However, in the C22 the proportion of PE is higher from 5 weeks of age compared to the corresponding WT (Fig. 4G, H). The LPE concentration is significantly higher in the C3 at 12 weeks and at 3, 5 and 7 weeks in the C22 compared to the control (Supplementary Fig. S12A, B). Within the PE lipid class, we observed a consistent reduction of 18:1 PE, while 18:0 and 16:0 were increased in the C3 and the C22 (Supplementary Fig. S12C, D). The LPE lipid species showed an increase in poly-saturated fatty acids, especially at 3 weeks of age (Supplementary Fig. S12E, F).

Although the proportion of phosphatidic acid (PA) in the lipidome of the sciatic nerve is low, the concentration of PA is decreased in the C3 at 3, 5 and 12 weeks, but higher in the C22 at 3, 7, and 12 weeks compared to corresponding WT mice (Supplementary Fig. S13A, B). The PA lipid species shift towards shorter fatty acids for both the C3 and the C22 compared to the corresponding wild types (Supplementary Fig. S13C, D). The conversion from PA to CDP-diacylglycerol (CDP-DG) occurs through CDP-DG synthase (CDP-DGS), encoded by CDP-diacylglycerol synthase 1 and 2 (*Cds1* and *Cds2*), of which *Cds2* is downregulated in the C3 and C22 (Fig. 4A, B). Subsequently, CDP-DG can be converted to phosphatidylinositol (PI). In the C3, we observe a lower percentage of PI at 7 weeks of age in the sciatic nerve compared to the control, while C22 have a higher percentage at 3 and 5 weeks of age (Fig. 4I, J). However, the synthesis of PI is downregulated in both the C3 and C22 mice compared to WT (Fig. 4A, B).

In summary, *PMP22* overexpression causes a consistent downregulation in phospholipid concentration with a proportional shift towards PC and LPC. Moreover, there are distinct changes in fatty acid composition with a notable increase in LPE with a shift to poly-unsaturated lipids and PS and PE with an increased level of unsaturated fatty acids, already present from 3 weeks of age. Combined with the altered transcriptomics, *PMP22* overexpression causes changes in the Lands Pathway, a pathway important in the modulation of the fatty acids of the phospholipids, and the Kennedy pathway (32).

### Sphingomyelin and ceramide concentrations and synthesis are severely affected in CMT1A mouse models

Sphingolipids (SLs) contain a sphingoid base (sphingosine (d18:1) or sphinganine (d18:0)) and a fatty acid N-acyl chain. SM and CER are both SLs and are metabolically interconnected. They play crucial roles as signaling lipids for myelination and contribute significantly to lipid raft formation (33, 34). The N-acyl chain composition affects the properties of CER and SM, among which is their preference for different phases, surface topography, and transmembrane binding capacity (34).

**Fig. 5:**
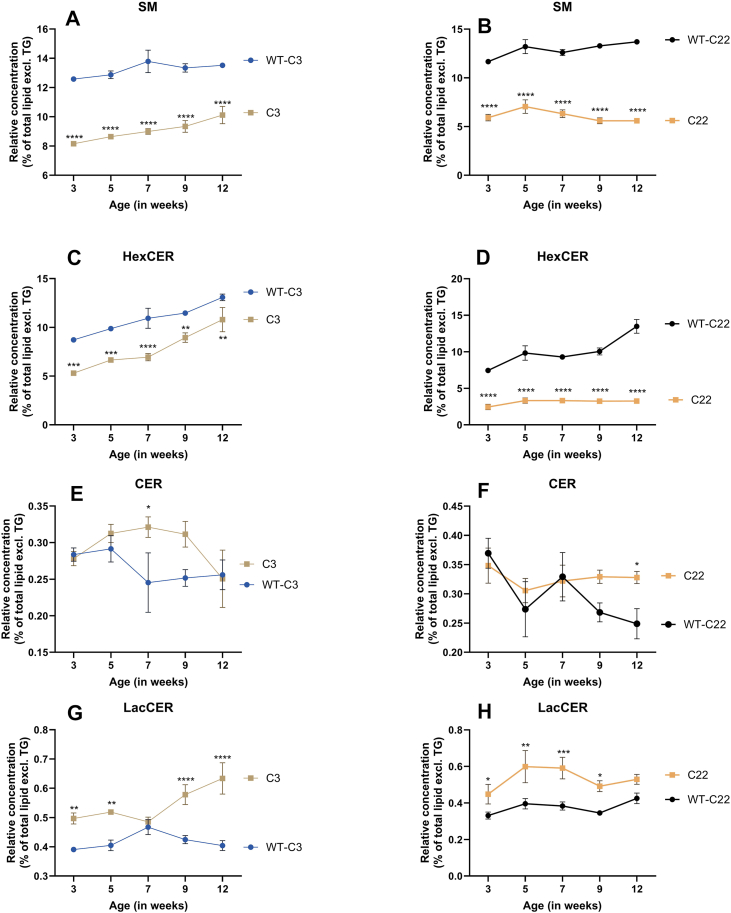
Alteration in sphingolipid concentration present in the sciatic nerve of CMT1A mice. A-H: The concentration of the sphingolipids; ceramide (CER), sphingomyelin (SM), hexosylceramide (HexCer) and lactosylceramide (LacCer), per mg protein divided by the lipidome (excluding TG) of the C3 and C22 and the corresponding wildtypes at 5 time points. Data is visualized as the mean ± SEM, analysis was preformed using a two-way ANOVA with a Tuckey post hoc analysis (∗*P* < 0.05, ∗∗*P* < 0.002, ∗∗∗*P* < 0.0002, ∗∗∗∗*P* < 0.0001).

For the C3 and C22 mouse models, the concentration of CER and SM is lower at all ages (Supplementary Fig. S14A–D). Relative SM and hexosylceramide (HexCer) concentrations remain consistently lower in both the C3 and C22 compared to WT (Fig. 5A–D). However, the relative CER concentration corrected for the total lipidome is higher in C3 at 7 weeks and in the C22 at 12 weeks of age compared to WT (Fig. 5E, F). The relative lactosylceramide (LacCER) concentration is higher in the C3, except for 7 weeks of age (Fig. 5G), while for the C22 mice, the concentration is higher until 12 weeks of age (Fig. 5H).

**Fig. 6:**
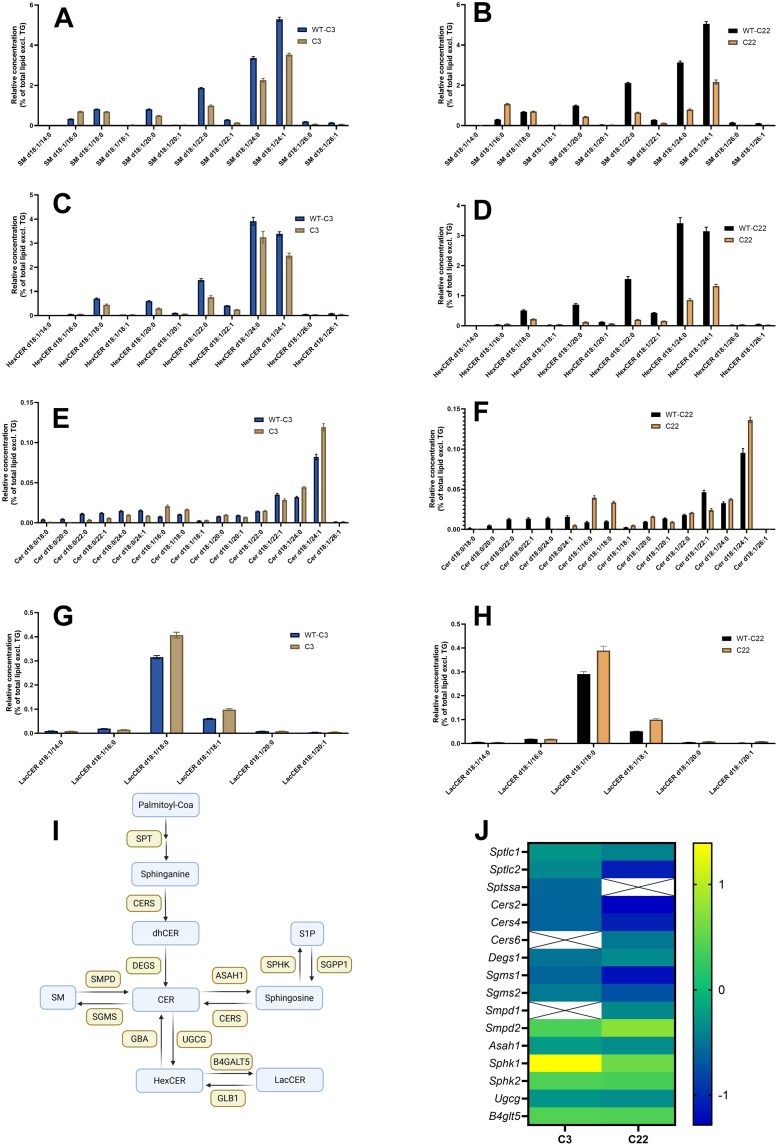
Proportional shift in sphingolipid species present in the sciatic nerve of CMT1A mice. A-H: Visualized is the concentration of the different lipid species of the sphingolipids; sphingomyelin (SM), hexosylceramide (HexCer), ceramide (CER) and lactosylceramide (LacCer), per mg protein divided by the lipidome (excluding TG) of the C3 and C22 and the corresponding wildtypes at 5 time points. concentration. Data is visualized as the mean ± SEM. I & J: A schematic overview of the sphingolipid (SL) synthesis pathway and important genes involved in this pathway. The Heatmap shows the log2(fold change) of differentially expressed genes in the CMT1A mouse models, important in the SL synthesis pathway. ASAH1, N-Acylsphingosine Amidohydrolase 1; B4GALT5, Beta-1,4-Galactosyltransferase 5; CERS2/4/6, Ceramide Synthase2/4/6; DEGS1, Delta 4-Desaturase, Sphingolipid 1; SGMS1/2, Sphingomyelin Synthase half; SMPD1/2, Sphingomyelin Phosphodiesterase half; SPHK1/2, Sphingosine Kinase half; SPTLC1/2, Serine Palmitoyltransferase Long Chain Base Subunit half; SPTSSA, Serine Palmitoyltransferase Small Subunit A; UGCG, UDP-Glucose Ceramide Glucosyltransferase. Figure 6I was created in https://BioRender.com.

The reduced concentration of SM can be contributed to lower concentrations of; SM d18:1/22:0, SM d18:1/24:0, and SM d18:1/24:1. Contrary to this, the concentration of SM d18:1/16:0 is increased in both the C3 and C22 (Fig. 6A, B). The HexCER species are all reduced in concentration, with no specific changes in type of fatty acid (Fig. 6C, D). Moreover, CER d18:1/16:0 and CER d18:1/18:0 levels are elevated, with the most pronounced effect observed in the C22. Notably, the CER d18:0 (sphinganine) variants are almost absent in the C22 mice and significantly lower in the C3 (Fig. 6E, F). The increase in relative LacCER concentration is primarily attributed to the rise in LacCER 18:1/18:1 and LacCER18:1/18:0 (Fig. 6G, H).

Combining these observations, it would suggest that SLs with long-chain fatty acids are more impacted by *PMP22* overexpression and a shift towards SLs with shorter fatty acids occurs. Contrary to this, there is a proportional increase of CERd18:1/24:1 in the C3 and C22 mice compared to the corresponding WT, but these represent a really small fraction of the total lipidome (Fig. 6E, F).

#### The de novo synthesis of SM is downregulated in CMT1A mouse models, with specific CER species

The transcriptome analysis corroborated the observed decrease in SM and CER levels with clear changes in the SL metabolism. Transcriptomics data analyzed in IPA show that both synthesis, transport and modification of sphingolipids are massively impacted due to *PMP22* overexpression, with a clear effect of *PMP22* copy number (Table 1). Underlying these changes are interesting mechanisms via which the observed changes in the SL lipid species present come to show. The formation of sphinganine, a rate-limiting step in de novo synthesis of SM and CER, is significantly impaired in the C3 and C22. For sphinganine synthesis, palmitoyl-CoA undergoes conversion to 3-Ketosphinganine through the action of Serine Palmitoyltransferase (SPT). Two subunits of SPT, namely Serine Palmitoyltransferase Long Chain Base Subunit 1 (*Sptlc1*) and 2 (*Sptlc2*), are downregulated in the C3 and C22 (Fig. 6I, J). The Serine Palmitoyltransferase Small Subunit A (*Sptssa*) expression level is not differentially expressed in the C22 but decreased in the C3 (Fig. 6J). Sphinganine is subsequently converted by Ceramide Synthases (CERS) to dhCER (Fig. 6I) (35, 36). CERS has several variants, each exhibiting a preference for distinct acyl chain length. The transcription levels of *Cers2* and *Cers4*, both preferentially synthesize very long acyl chains (C22-24 and C18-22, respectively), are downregulated in the C3 and C22 mice (Fig. 6J). In contrast, *Cers1*, responsible for synthesizing C18 ceramides, is not differentially expressed in the C3 and C22. While *Cers6*, responsible for synthesizing C14-C16 ceramide, is down regulated only in C22 (Fig. 6J). Showing a mechanism via which the proportional changes in fatty acid length in SLs occurs. When looking into *Cers* expression over time, we see that *Cers6* expression correlates positively with time in the WT, while *Cers2* is negatively correlated, suggesting that the fatty acid shift in CER also occurs during aging (Supplementary Fig. S15).

The conversion of dhCER to CER is mediated by desaturase enzyme (*Degs1*), which exhibits decreased expression levels in both the C3 and C22. Furthermore, the conversion of CER to SM and HexCER is also attenuated (Fig. 6J). However, the conversion from SM to CER demonstrates a heterogeneous pattern. Notably, acid sphingomyelinase, encoded by the gene Sphingomyelin Phosphodiesterase 1 (*Smpd1*), is downregulated in the C22, while neutral sphingomyelinase, encoded by *Smpd2*, is upregulated in C3 and C22 mice. Acid sphingomyelinase predominantly facilitates lysosomal breakdown of SM, whereas neutral sphingomyelinase catalyzes SM directly at the plasma membrane (37). Ultimately, membrane lipids are destined for degradation in lysosomes through acid ceramidases (encoded by *Asah1*) into sphingosine. The degradation of sphingosine-by-sphingosine kinase (*Sphk*) into sphingosine-1-phosphate is increased in C3 and C22. Conversely, the reintegration of sphingosine via CERS into SL synthesis is reduced (Fig. 6J).

### Plasmalogen levels are severely reduced and accompanied by a proportional shift in CMT1A mouse models

**Fig. 7:**
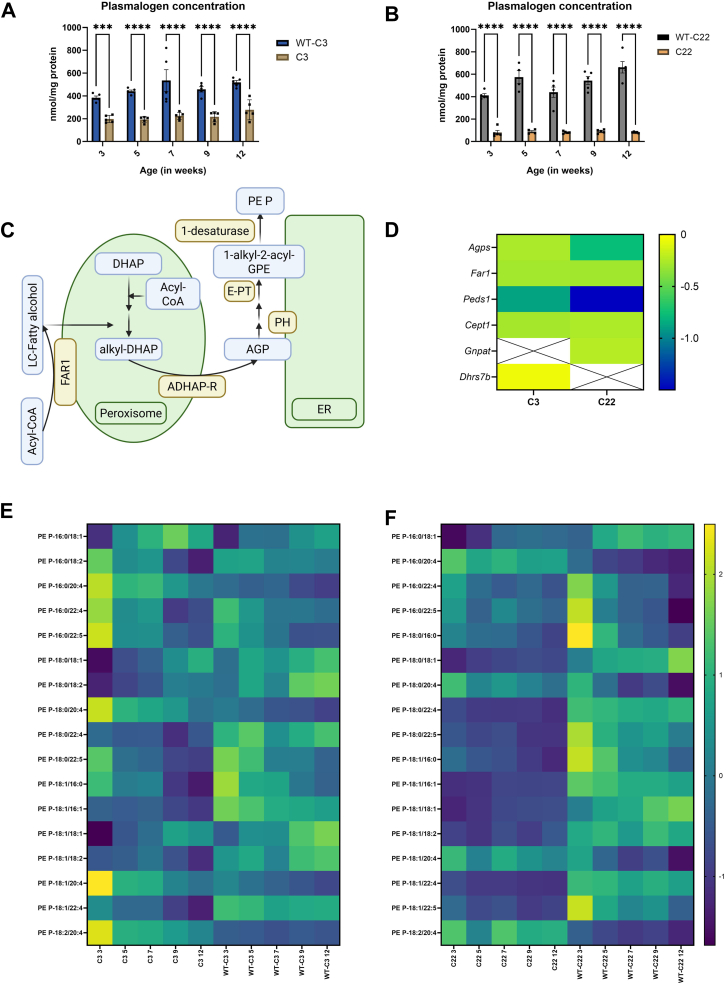
Reduced plasmalogen concentration with clear reduced synthesis of plasmalogens in the sciatic nerve of CMT1A mice. A & B: The plasmalogen (PE P-) concentration per mg protein of the C3 and C22 and the corresponding wildtypes at the 5 time points. Data is visualized as the mean ± SEM, analysis was performed using a two-way ANOVA with a Tuckey post hoc analysis (∗∗∗*P* < 0.0002, ∗∗∗∗*P* < 0.0001). C: A schematic overview of the de novo synthesis of plasmalogens. Figure was created in https://BioRender.com. D: A heatmap of differentially expressed genes depicting the log2(fold change) between the C3 compared to WT-C3 and C22 compared to WT-C22. We observed a consistent reduced expression of genes involved in plasmalogen synthesis in the C3 and C22 mice compared to corresponding controls. E & F: Heatmaps of plasmalogen species over time in the sciatic nerve of the C3 and C22 and their controls over time. The heatmap is visualized with the z-score based on the mean after normalization for total lipid concentration. ADHAP-R, acyl/alkyl DHAP reductase; AGP, 1-akyl-2-lyso-sn-glycero-3-phosphate; Agps, Alkylglycerone Phosphate Synthase; Cept1, Choline/Ethanolamine Phosphotransferase 1; DHAP, droxyacetone phosphate; Dhrs7, Dehydrogenase/Reductase 7; E-PT, ethanolamine-phosphotransferase; ER, endoplasmic reticulum; Far1, Fatty Acyl-CoA Reductase 1; Gnpat, Glycerophosphate O-Acyltransferase; Peds1, Plasmanyl ethanolamine desaturase 1; Peds1, Plasmanylethanolamine Desaturase 1; PH, phosphohydrolase.

Plasmalogens have been identified as crucial for myelinating Schwann cells and neuronal survival following injury (38, 39). Plasmalogens can act as signaling lipids by participating in the MAPK/ERK and PI3K/AKT pathways (40). In the C3 and C22 the plasmalogen concentration is significantly lower compared to the WTs (Supplementary Figs. S16 and Fig. 7A, B). The transcriptome supports the observed reduction in plasmalogen levels, with lower expression of genes important for the synthesis of plasmalogens (e.g. Fatty acyl-CoA reductase 1 (*Far1*), Plasmanyl ethanolamine desaturase 1 (*Peds1*), Alkylglycerone Phosphate Synthase (*Agps*), choline/ethanolamine phosphotransferase (*Cept1*) (Fig. 7C, D). Additionally, Glyceronephosphate O-acyltransferase (encoded by the gene *Gnpat*), one of the rate-limiting enzymes in plasmalogen synthesis, is downregulated in the C22 (Fig. 7C, D). Conversely, *Dhrs7b*, a gene encoding for acyl/alkyl DHAP reductase (*Adhapr*), which catalyzes acyl-DHAP to form 1-alkyl-2-lyso-*sn*-glycero-3-phosphate (AGP), is downregulated in C3 mice but not significantly altered C22 (Fig. 7D) (40, 41).

Furthermore, the proportional shift in the plasmalogens over time shows a consistently lower concentration in two abundant plasmalogen species (PE P-18:1/18:1 and PE P-18:0/18:1), in C22 and C3 compared to their WT counterparts (Fig. 7E, F). These two plasmalogens already account for approximately 40%–60% of the total plasmalogen content in the WT. However, the proportion of PE P-16:0/18:1 is higher in C3 mice compared to the WT. Additionally, plasmalogens containing arachidonic acid (20:4), including PE P-18:1/20:4, PE P-18:0/20:4, PE P-16:0/20:4, and PE P-18:2/20:4, are relatively more abundant in the C3 and C22 mice compared to the corresponding WT (Fig. 7E, F). Notably, the concentrations of these three plasmalogen subtypes with oleic acid (18:1) are positively correlated with age in all mice. While the subtypes with FA 20:4 (PE P-18:1/20:4, PE P-18:0/20:4, and PE P-16:0/20:4) show a consistent negative correlation with age in all groups (Fig. 7E, F).

### Initiation and regulation of myelination are hampered due to PMP22 overexpression

The transcriptome of the C3 and C22 mice, analyzed in IPA, shows a clear decrease in the Myelination signaling pathway in both C3 and C22 (Table 2). Moreover, signaling pathways in regulating myelin formation and maturation, ERK/MAPK and Protein phosphatidylinositol 3-kinases/kinase B (also known as PI3K/AKT), have significant negative z-scores in both the C3 and the C22 compared to WT (Table 2). This inactivation is associated with hypomyelination. The downregulation of these pathways is combined with the downregulation of mTOR signaling, which is downstream of the ERK/MAPK and PI3K/AKT signaling pathways. The phosphatase and tensin homolog (PTEN) signaling pathway, is upregulated in both the C3 and C22. As PTEN is a direct negative modulator of the PI3K/AKT signaling pathway, these observations are all intertwined. Moreover, we observe an increased expression in the autophagy pathway and the Coordinated Lysosomal Expression and Regulation (CLEAR) signaling pathway in the C3, while in the C22 both these pathways have a negative z-score (Table 2).

**Table 2:** Differential expression in pathways important for myelination and lipid homeostasis in the sciatic nerves of CMT1A mice models compared to controls

Pathways	C3 versus WT-C3		C22 versus WT-C22	
	-log(*P*-value)	Z-score	-log(*P*-value)	Z-score
Myelination Signaling Pathway	14.6	−0.33	18.3	−1.96
ERK/MAPK Signaling	4.17	−0.34	7.84	−1.09
PI3K/AKT Signaling	2.04	−1.04	2.1	−0.38
mTOR Signaling	3.19	−1.4	2.65	−1.27
PTEN Signaling	4.92	0.962	9.27	1.98
Autophagy	3.89	0.82	3.64	−0.56
CLEAR Signaling Pathway	3.59	1.73	6.33	−0.23

The bulk RNA sequencing data of the sciatic nerve of C3 and C22 is analyzed with ingenuity pathway analysis. A significant downregulation in the C3 and C22 compared to control is observed in multiple pathways: Myelination Signaling Pathway, ERK/MAPK Signaling, PI3K/AKT Signaling, mTOR Signaling. While the following pathways are upregulated in the C3 compared to controls: PTEN Signaling, Autophagy, Coordinated Lysosomal Expression and Regulation (CLEAR) Signaling Pathway.

To conclude, pathway analysis shows reduced myelination, with reduced expression in myelination signaling pathways. These are combined with altered autophagy and lysosomal regulation, which are differently affected in the C3 and C22 mice models.

## Discussion

Myelination is fundamental for signal propagation and Schwann cell-axon interaction. In CMT1A, demyelination of the peripheral nerves is well-characterized, ultimately resulting in axonal degeneration. In the current study, we analyzed the lipidome and transcriptome of two mouse models of CMT1A, the C22 and the C3, across five different timepoints. Our findings reveal a consistent depletion in myelin-associated lipids, including cholesterol, sphingomyelin, phospholipids and plasmalogens, in both CMT1A mouse models compared to controls. We observed a dosage effect of *PMP22* copy number, with the C22 model, harboring a higher *PMP22* dosage, exhibiting more pronounced alterations in both lipidomic and transcriptomic landscapes compared to the C3 model. This is in line with the severity of the neurological phenotype.

For proper myelin formation, not only lipid concentration itself but also the relative proportions of lipids and lipid species are crucial (42, 43, 44). Our analysis of the C3 and C22 models extends our previous work showing decreased cholesterol synthesis (15). This study shows that these changes result in a distinct reduction of sterol concentrations. Lathosterol, cholesterol, and cholestanol concentrations are reduced in the C3 and C22, with concentrations in C22 being more impacted than in the C3 mice. The CE concentrations were elevated in both the C22 and C3 compared to the WT mice, which has previously been associated with demyelination (45). In addition, we observed progressive accumulation in CE levels over time in the C22, whereas CE concentration declines over time in the C3. This temporal normalization of CE concentration is linked to remyelination, with CE clearance shown to correlate with remyelination. In contrast, in chronic demyelination of the spinal cord the CE concentration stays elevated (45). Suggesting that in the C22 the demyelination is more chronic and severe compared to the C3, where there might be partial remyelination. CE are formed to transport and store cholesterol in the cell and protect the cell from potential adverse effects of oxysterols (46, 47). This increase in CE while cholesterol synthesis is hampered can also be viewed as a more generic neurodegenerative phenotype, as it is also observed in AD and PD (48, 49). However, how this phenotype comes to show differs greatly. We hypothesize that in the case of CMT1A the altered sterol synthesis and concentrations is a result of the link between PMP22 and cholesterol homeostasis. One argument for this is that PMP22 contains CRAC and CARC motifs, enabling PMP22 to directly bind cholesterol (10, 11). Moreover, cholesterol synthesis, based on RNA expression levels, was increased by reducing PMP22 levels with antisense oligonucleotides in CMT1A animal models, strengthening the link between *Pmp22* expression levels and cholesterol homeostasis (50). Additionally, a study in PMP22 knockout mice and fibroblasts from CMT1A patients has shown that PMP22 levels are important for proper ABCA1-mediated cholesterol efflux (12). It has also been shown, in rat Schwann cells, that drug induced increases in CE concentration results in increased *Pmp22* levels (13). We further hypothesize that CE accumulation may result from impaired incorporation of cholesterol into the membrane, leading to its storage in esterified form. Which is supported by the observation that ABCA1 is upregulated in our CMT1A mouse models. Combining this with the alterations in CLEAR signaling and autophagy pathways, we argue that there is an interdependence between PMP22 and cholesterol homeostasis. Suggesting that the link between *PMP22* expression levels and cholesterol could be a potential driver of CMT1A pathology. Although the precise mechanisms underlying these shifts remain to be elucidated, our data indicate that modulation of cholesterol synthesis represents a promising avenue for therapeutic interventions in CMT1A.

In addition to the changes in sterol concentration, we show that from 3 weeks of age, the C3 mice have a lower phospholipid and SM concentration. With additional proportional shifts, such as the increase in PC at the expense of PE. A previous study in a CMT1A rat model showed similar changes at P5 and P10 (51), with PC and PE being relatively increased. The reduced SM and phospholipid concentrations were also reported in Prior, Silva (15) in both C3 mice (at 5 weeks of age) and in iPSC-derived Schwann cell precursors of CMT1A patients. We conclude that the change in phospholipids and SM is consistent across various models of CMT1A and from a very early stage of development. Notably, the reduced concentrations in SLs and plasmalogens are combined with shifts in fatty acids attached to the acyl group of these lipids. These fatty acids play a vital role in determining the preference for a specific phase, surface topography, and transmembrane binding properties of these lipids (34, 52). The proportion of SM containing shorter and more saturated fatty acids is increased in C3 and C22. Conversely, the plasmalogen species exhibited a proportional increase in FA 20:4, while the plasmalogens with FA 18:1 were reduced in both CMT1A mouse models. These alterations in plasmalogens and SM species influence myelin properties, leading to distinct differences in myelination between CMT1A and control groups. The concentration of plasmalogens and SM is closely linked to cholesterol transport, transmembrane function, and the efficiency of vesicular membrane fusion (53). Interestingly, plasmalogen levels in the membrane modulate the PI3K/AKT signaling. Deficiency of plasmalogen levels is suggested to inhibit PI3K/AKT signaling resulting in altered mTOR signaling and reduced myelination (54). We observed both pathways being downregulated in the C3 and C22. The observed changes in FA composition and the overall reduced concentration of plasmalogens are also observed in peroxisome biogenesis disorders, such as Zellweger spectrum disorders (ZSDs) and rhizomelic chondrodysplasia punctata (RCDP) type 1 (55). In patients with ZSD and RCDP, defects of myelination are observed (56, 57). The lipidomics data would suggest that the peroxisomal functioning is hampered, although this is not fully supported by our RNA-seq data, making plasmalogen synthesis and peroxisomal functioning very interesting targets to mitigate the potential negative effects of demyelination.

We also observed changes in FA composition within SM and CER lipid classes. These changes may reflect alterations in the intracellular transport of newly synthesized CER from the ER to the Golgi, where it can be converted into SM. Typically, this process occurs either via vesicles or via ceramide transfer protein (CERT). CERT prefers the transport of CER with an acyl chain length less than 22 carbon atoms (58). As proposed by Prior, Silva (15), PMP22 overexpression may lead to difficulties in handling lipids and the clearance of lipid droplets. The absence of alternative transport routes causes dependence on the transport of CER via CERT. In CMT1A, this could contribute to the proportional changes in fatty acids available to synthesize SM, resulting in a shift toward SM with shorter carbon chains (33). Alterations in fatty acid composition, exemplified by elevated levels of lignoceric acid (24:0) and palmitic acid (16:0), have been implicated within the pathophysiological framework of multiple neurodegenerative disorders, including AD and PD (59, 60). An intriguing hypothesis is that for compact myelin formation, myelin proteins (e.g. PMP22, MBP, P2, and PLP) need to bind to phospholipids to stack lipid bilayers (61, 62, 63). The preference of myelin proteins for specific lipids and ordered membranes can vary depending on the presence of cholesterol and/or SM. Therefore, the changes in the lipid composition can both be a result and driver of dysmyelination (42, 62, 63). When *PMP22* expression is altered, this balance is disrupted, leading to fragile and dysfunctional myelin, which in turn causes the characteristic demyelinating phenotype. It remains important to note that lipid homeostasis is also post-transcriptionally regulated, gaining insight into this post-transcriptional role of PMP22 in lipid metabolism would be relevant for understanding how the observed changes come to show in CMT1A.

In conclusion, our findings highlight a crucial role for SM, plasmalogen and cholesterol metabolism in the pathogenesis and progression of CMT1A. Transcriptomics showed dysregulated lipid homeostasis, which were dependent on *PMP22* copy number, leading to alterations in the lipid species present in the lipidome. These findings suggest multiple potential pathways by which *PMP22* overexpression can drive dysmyelination and demyelination and show potential ways to intervene in the disease progression. We propose opportunities for dietary interventions, specifically boosting the plasmalogen levels, to improve and support myelination in CMT1A. Previous studies on lipid enriched diets in CMT1A models showed promising results (14, 17). However, with the knowledge presented here, we hypothesize that plasmalogen supplementation with consideration of fatty acid composition would yield better results. As plasmalogens form a major component of the membrane and could regulate the cholesterol homeostasis (42, 64). Moreover, plasmalogen replacement therapy, a strategy to improve plasmalogen levels by supplementation of small molecules, has been effective in multiple pathologies (e.g., AD and PD) and increases nerve conduction with minimal negative effects (40, 65).

## Data availability

The data of C3 and C3-WT without TG can be interactively viewed via; https://cpm-lumc.shinyapps.io/soda-light/?experimentId=NLA_920 and for C22 and C22-WT without TG via https://cpm-lumc.shinyapps.io/soda-light/?experimentId=NLA_918. The RNA-seq data is available in the NCBI's gene expression omnibus (GEO) under number GSE312450. Data underlying this article will be shared on reasonable request to the corresponding author.

## Supplemental data

This article contains supplemental data.

## Conflict of interest

The authors declare that they do not have any conflicts of interest with the content of this article.
