# Supplementary material for: Longitudinal analysis of lipid changes in the sciatic nerve caused by overexpression of PMP22 in murine models of CMT1A
Source: J Lipid Res. 2026 Mar 11;67(4):101018. doi: 10.1016/j.jlr.2026.101018 (PMC13090589; doi:10.1016/j.jlr.2026.101018)
Supplement: Supplementary Figures [file mmc1.docx]

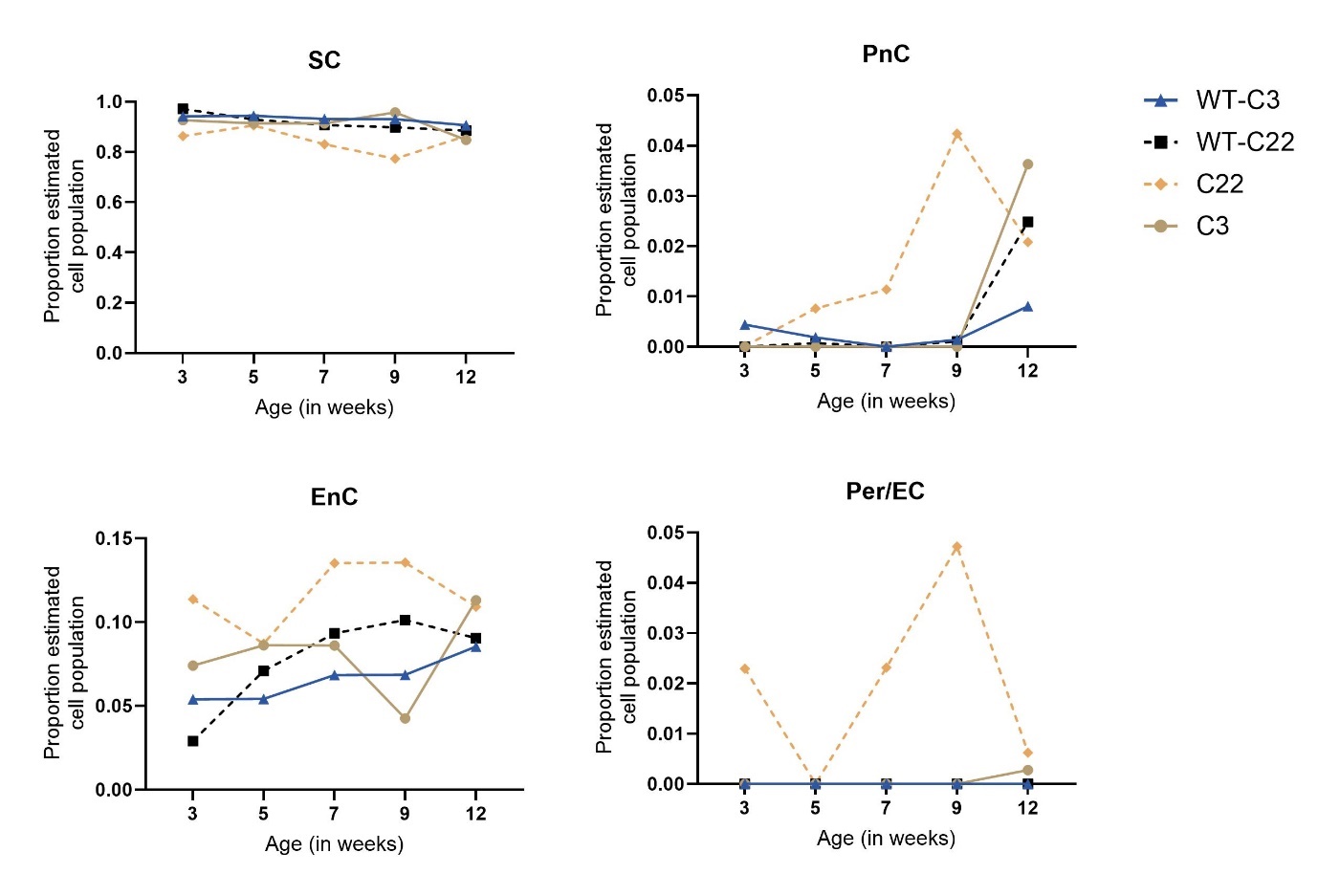


**Supplementary figure 1. Estimated cell population based on deconvolution of RNA-bulk sequencing of the sciatic nerve of CMT1A mouse models and the corresponding control over time.** A, B, C & D: It becomes apparent that Schwann cells (SC) are the major cell population in the axon with no major shift between the C3 and WT-C3 and the C22 compared to the WT-C22. There are relative lower amounts of perineurial (PnC), endoneurial (EnC) and pericytes and endothelial (Per and EC) cells in the sciatic nerve, based on the transcriptomics data. The estimated proportion per cell type is based on deconvolution of the RNA-bulk data set with MUSIC and a single nuclei data set from the sciatic nerve. Data is visualized as a single data point per timepoint.


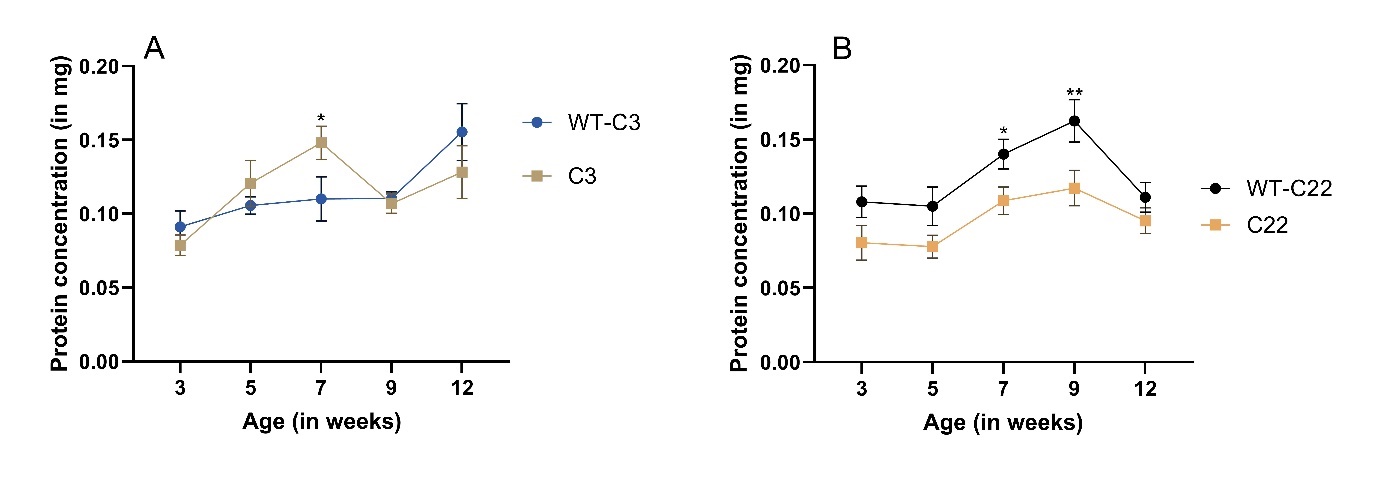


**Supplementary figure 2. Protein concentration of the sciatic nerve of CMT1A mouse models and the corresponding control over time used for normalization of the measured lipidome.** A: The protein concentration (in mg) the sciatic nerve of the C3 and the control (WT-C3) over time. B: The protein concentration (in mg) of the C22 and the corresponding control (WT-C22) over time. Data is visualized as the mean ± SEM. Analysis was preformed using a two-way ANOVA with a Tuckey post hoc analysis (*P<0.05 and **P < 0.002).


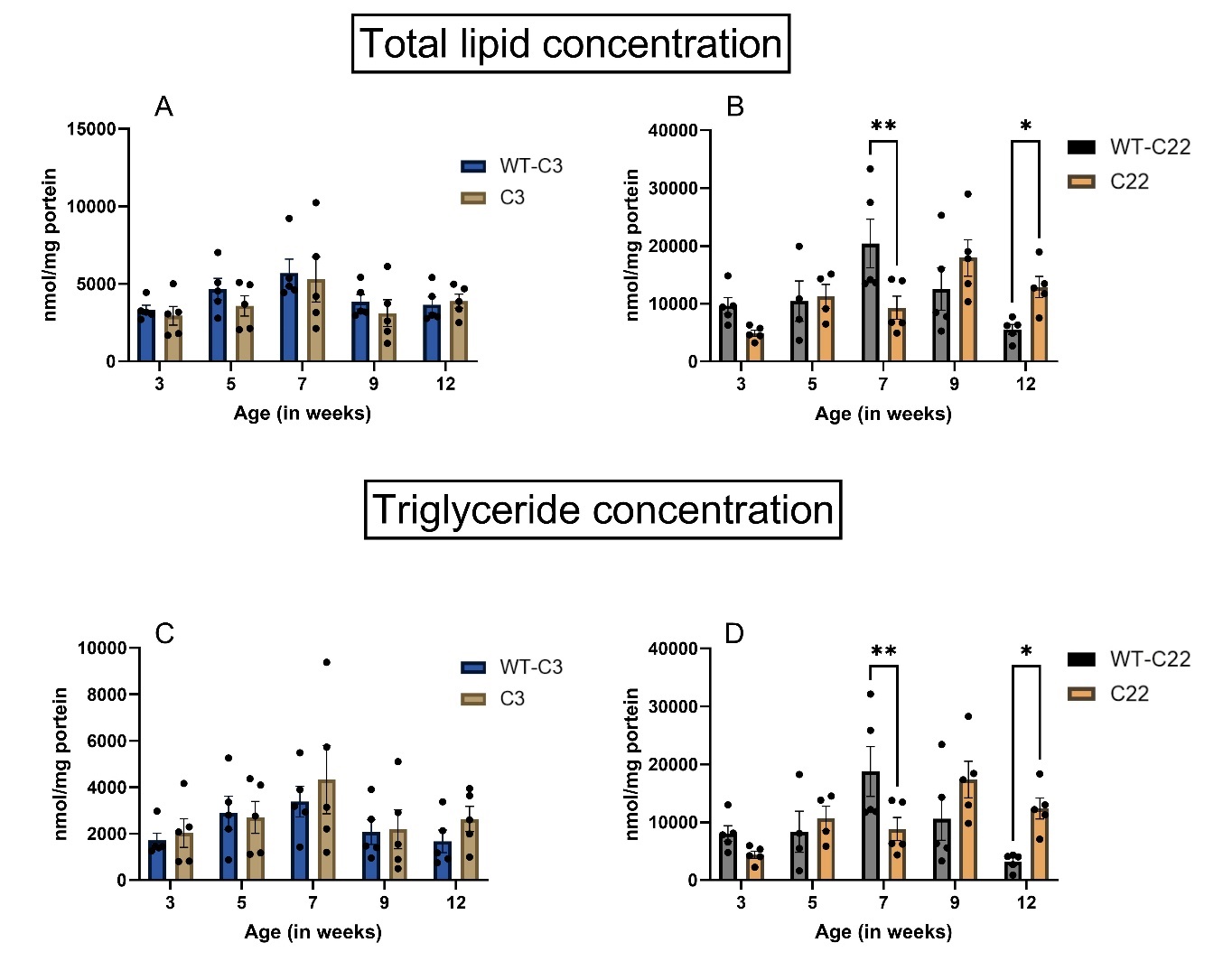


**Supplementary figure 3. Total and triglyceride concentration of lipids measured in the sciatic nerve of three different CMT1A. mouse models and their respective wild type control.** Visualized is the total lipid concentration (A & B) and the triglyceride concentration (C & D) measured by the Lipidized corrected for mg protein per sample of the sciatic nerve of C3, C22 and the corresponding controls at 5 different time points (3, 5, 7, 9 and 12 weeks of age). C&D: Data is visualized by means of a boxplot ± SEM, analysis was preformed using a two-way ANOVA with a Tuckey post hoc analysis (*P<0.05 and **P < 0.002).


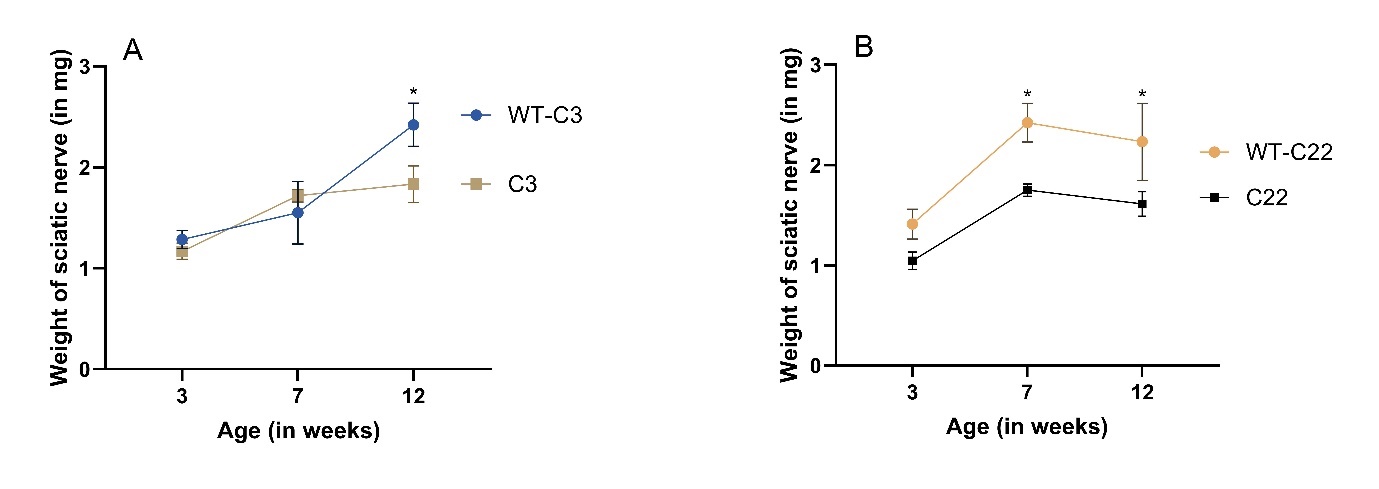


**Supplementary figure 4. The weight of the sciatic nerve of CMT1A mouse models and the corresponding control over time used for normalization of the cholesterol measurements.** A: The average weight (in mg) of the sciatic nerve of the C3 and the control (WT-C3). B: The weight of the sciatic nerve (in mg) of the C22 and the control (WT-C22). Data is visualized as the mean ± SEM. Analysis was preformed using a two-way ANOVA with a Tuckey post hoc analysis (*P<0.05).


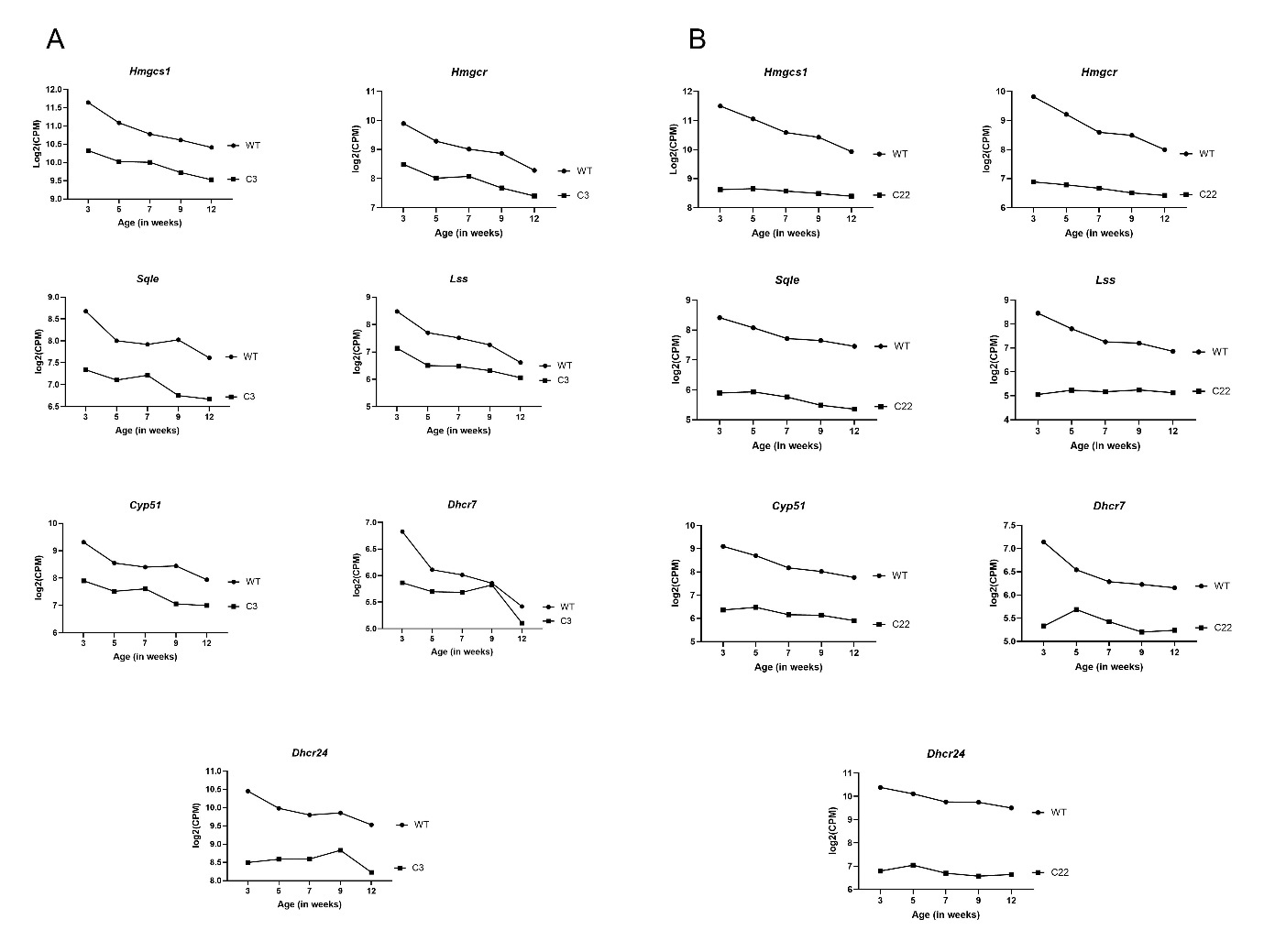


**Supplementary figure 5. Time course analysis of genes important for de novo cholesterol synthesis.** A & B: Visualized are expression levels of genes over time that play a pivotal role in cholesterol synthesis for C3 and the corresponding WT and C22 and the corresponding WT. The expression of all genes are negatively correlated with time in both the WT-C3 and WT-C22. *Hmgcs(1), 3-Hydroxy-3-Methylglutaryl-CoA Synthase 1; Hmgcr, 3-hydroxy-3-methylglutaryl CoA reductase; Sqle, Squalene Epoxidase; Lss, Lanosterol synthase; Cyp51, Lanosterol 14α-demethylase; Dhcr7, 7-Dehydrocholesterol Reductas; Dhcr24, 24-Dehydrocholesterol Reductase.*


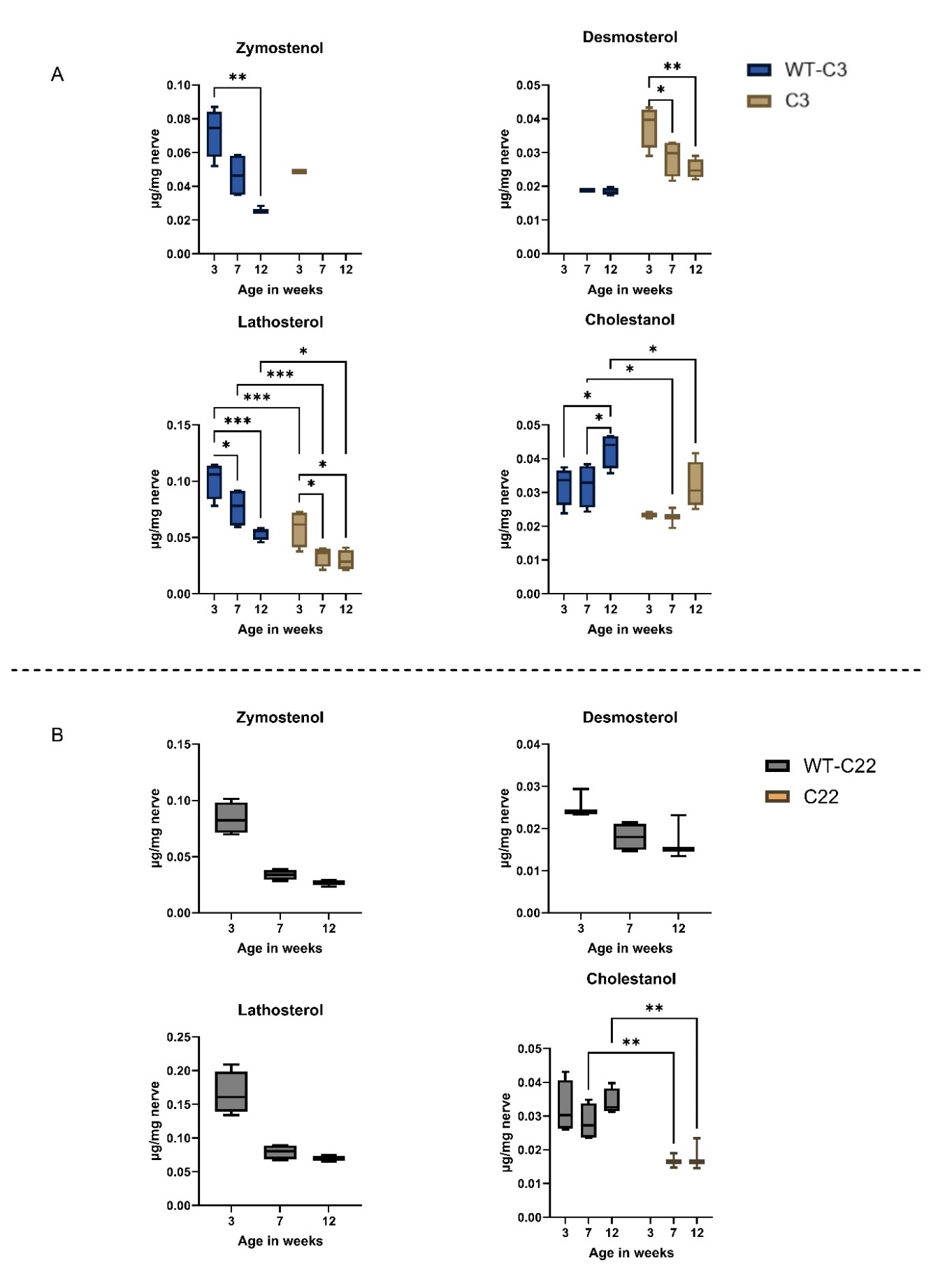


**Supplementary figure 6. Cholesterol precursors over time in the sciatic nerve of two different CMT1A mice models.** Concentration of the sterols measured in the sciatic nerves of the C3 (A), C22 (B) and the corresponding controls is normalized for the weight of the sciatic nerve in mg. We measured the concentrations of; zymostenol, desmosterol, lathosterol and cholestanol, at 3, 7 and 12 weeks of age. Data is visualized by means of a box plot ± SEM, analysis was preformed using a two-way ANOVA with a Tuckey post hoc analysis (*P<0.05, **P < 0.002, ***P<0.0002).


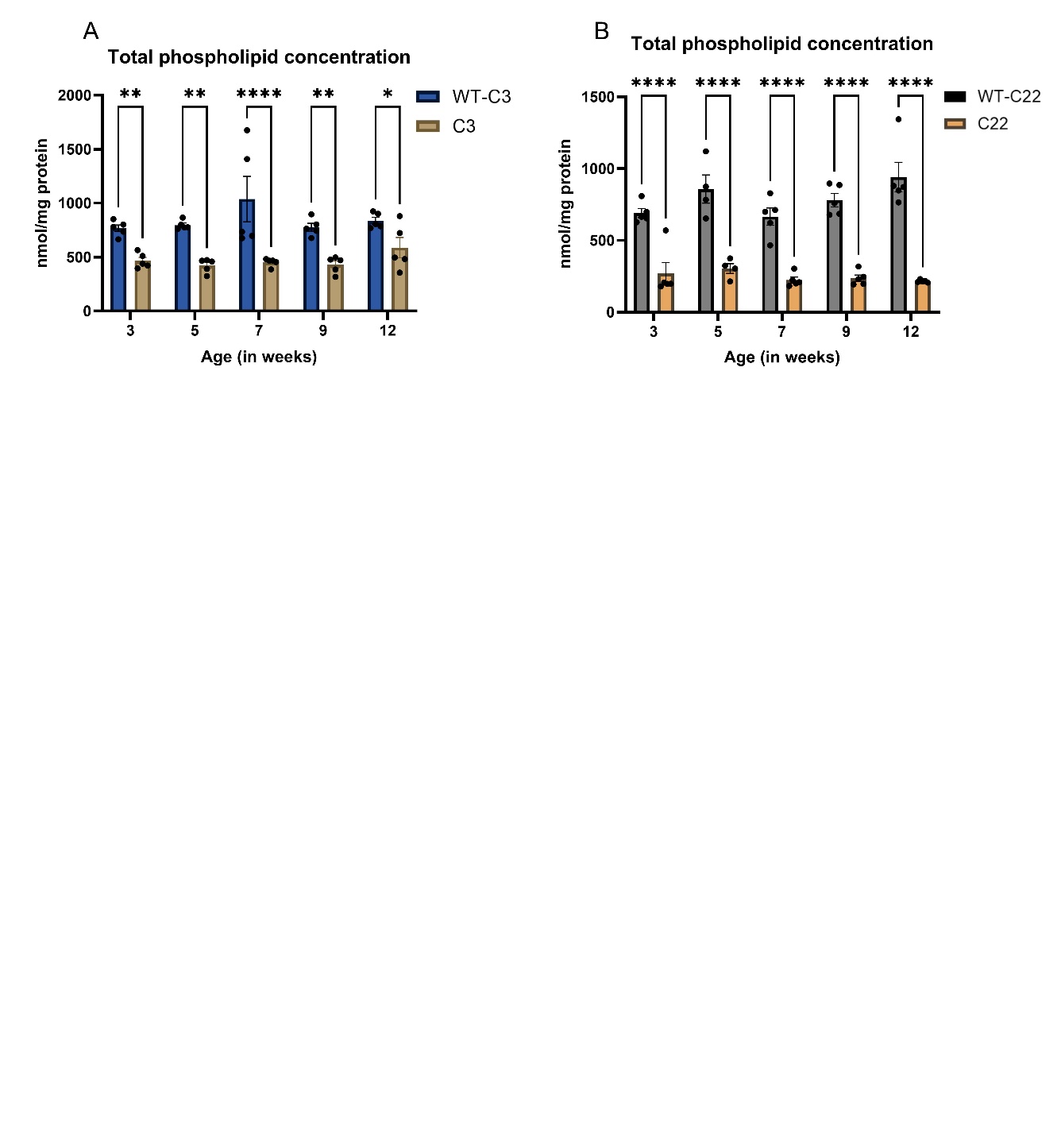


**Supplementary figure 7**. **Total phospholipid concentration in the lipidome of the sciatic of two different CMT1A mice models and the controls**. A & B: Total phospholipid concentration, calculated by cumulative quantification of: PC, PE, PA, PG, PI, PS, LPC and LPE. A: The concentration of phospholipids of the sciatic nerves corrected for protein concentration, is consistently lower in the C3 compared to the control (WT-C3). B: The concentration of phospholipids in the sciatic nerves corrected for protein concentration, is consistently lower in the C22 compared to the control (WT-C22). Data is visualized as the mean ± SEM, analysis was preformed using a two-way ANOVA with a Tuckey post hoc analysis (*P<0.05, **P < 0.002, **** P<0.0001).


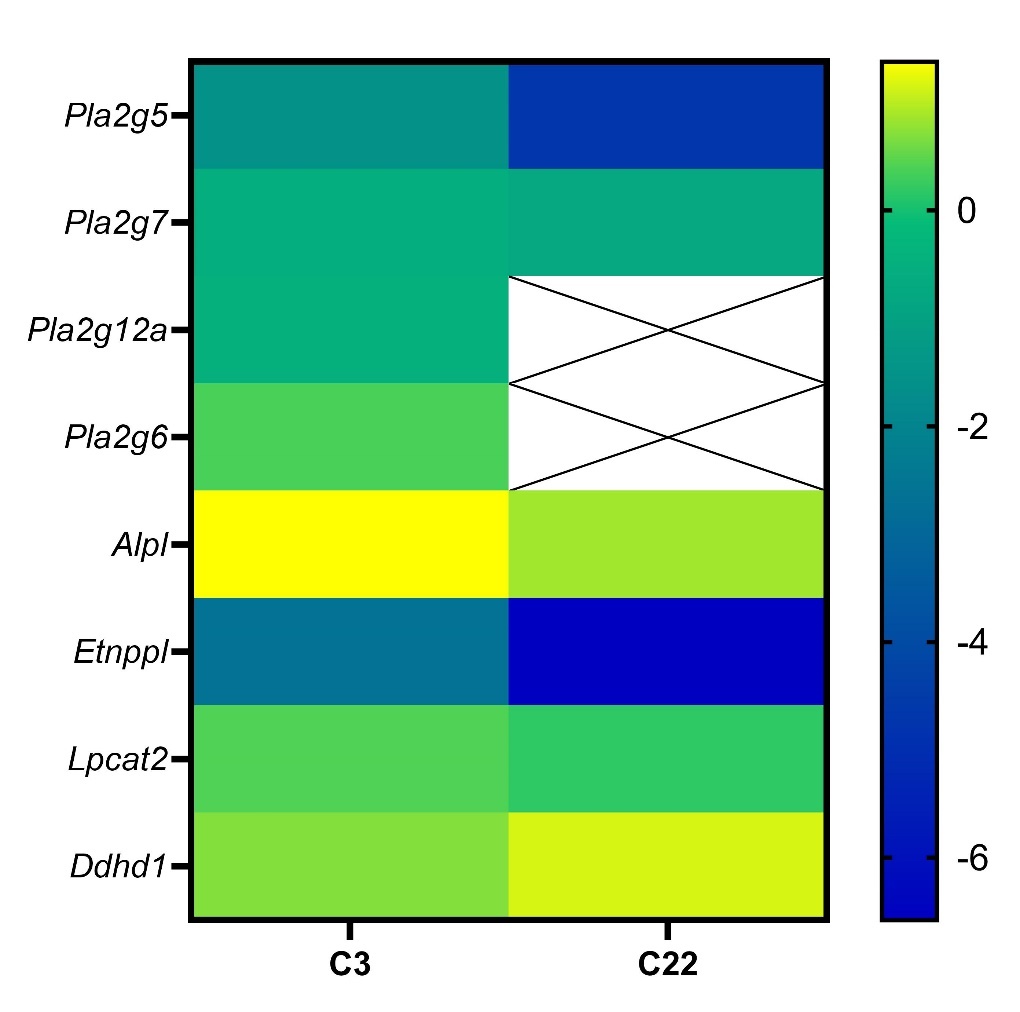


**Supplementary figure 8. Expression levels of genes important in the breakdown of phospholipids in two different CMT1A mouse models.** Visualized are the Log2(fold change) of expression levels of genes that play pivotal roles in the breakdown of different phospholipids in the C3 and C22 compared to the corresponding wild types. The genes shown are differentially expressed between the CMT1A mouse models and the corresponding controls, unless no color is shown, and thus no log2(fold change) value. *Pla2g5, Phospholipase A2 Group V; Pla2g7,* *Phospholipase A2 Group VII; PLA2G12a , Phospholipase A2 Group XIIA; ETNPPL, Ethanolamine-Phosphate Phospho-Lyase; LPCAT2, Lysophosphatidylcholine Acyltransferase 2; Dddh1, DDHD Domain Containing 1.*


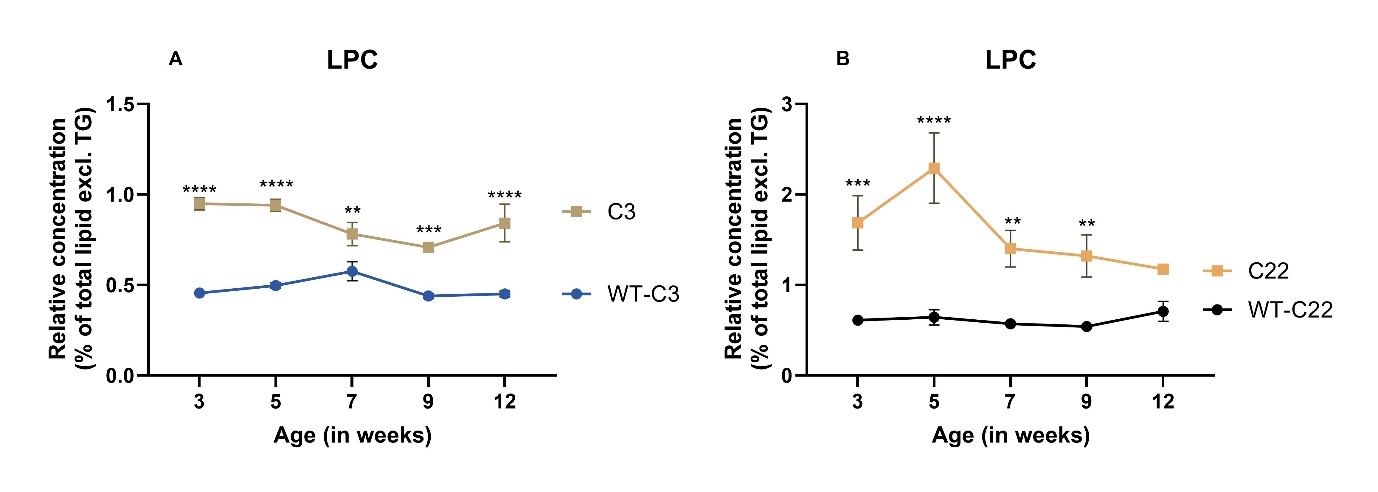


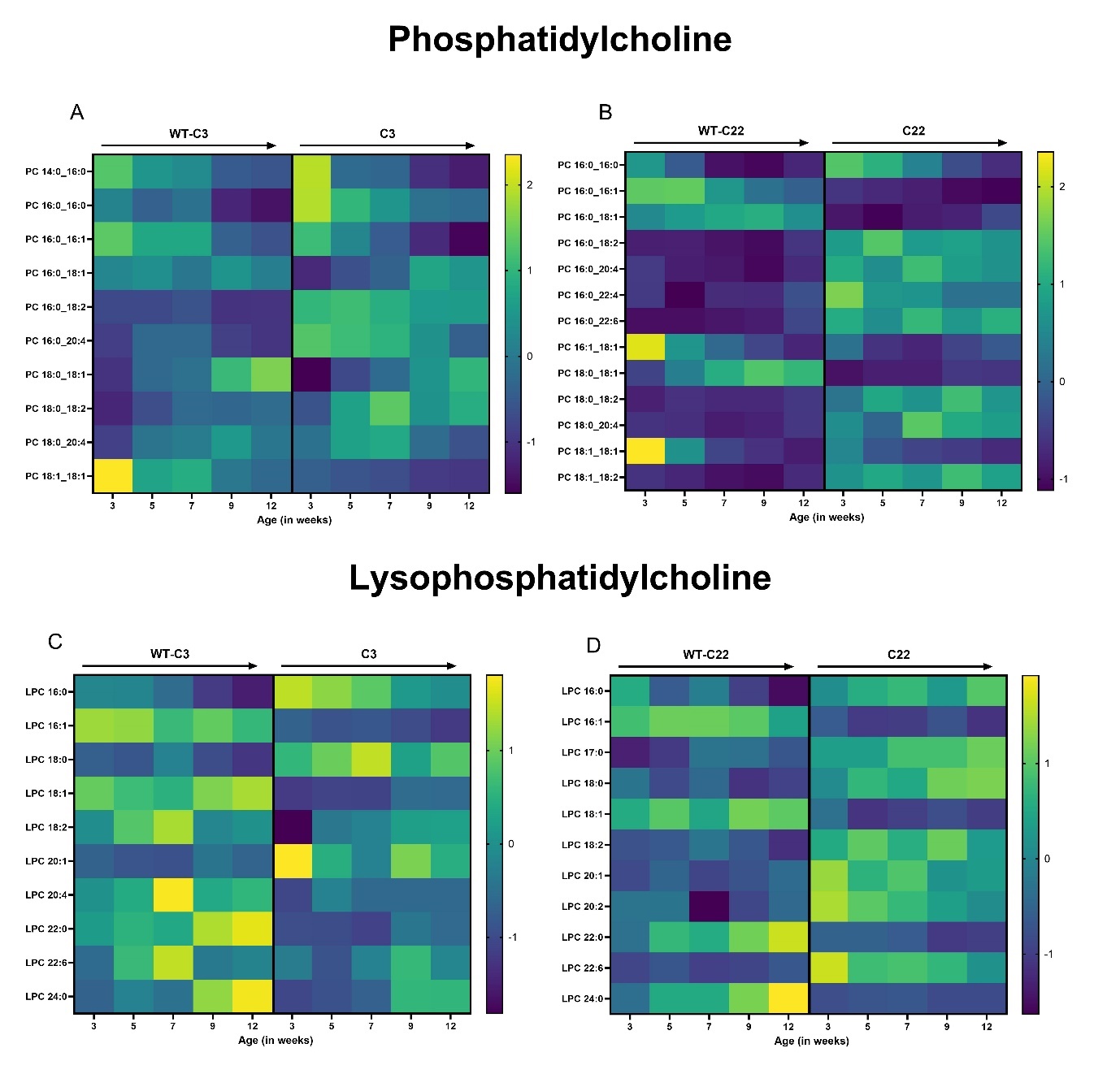


**Supplementary figure 9. Relative concentration of lysophosphatidylcholine (LPC) measured in the sciatic nerve of 2 different CMT1A mouse models.** A & B: LPC concentration per mg protein divided by the lipidome (excluding TG) over time in the sciatic nerve of the C3 (A) and C22 (B) and the corresponding controls. Data is visualized as the mean ± SEM, analysis was preformed using a two-way ANOVA with a Tuckey post hoc analysis (**P < 0.002, ***P<0.0002, **** P<0.0001).

**Supplementary figure 10. Heatmap of z-scores for relative abundance of phosphatidylcholine (PC) and lysophosphatidylcholine (LPC), measured in the sciatic nerve of 2 different CMT1A mouse models and controls over time.** The heatmap depicts z-scores of the lipid species present in each lipid class, normalized for the total concentration of that lipid class. Lipid species of which the average, the CMT1A model and WT combined, were below 1% of the concentration of the lipid class are not depicted in this heatmap. A shift from mono-unsaturated fatty acids towards ploy-unsaturated and saturated fatty acids for both the C3 (A & C) and the C22 (B & D) compared to the corresponding wildtypes in PC (A & B) and LPC (C & D) for the WT-C3, C3, WT-C22 and the C22 at 2, 5, 7, 9 and 12 weeks of age.


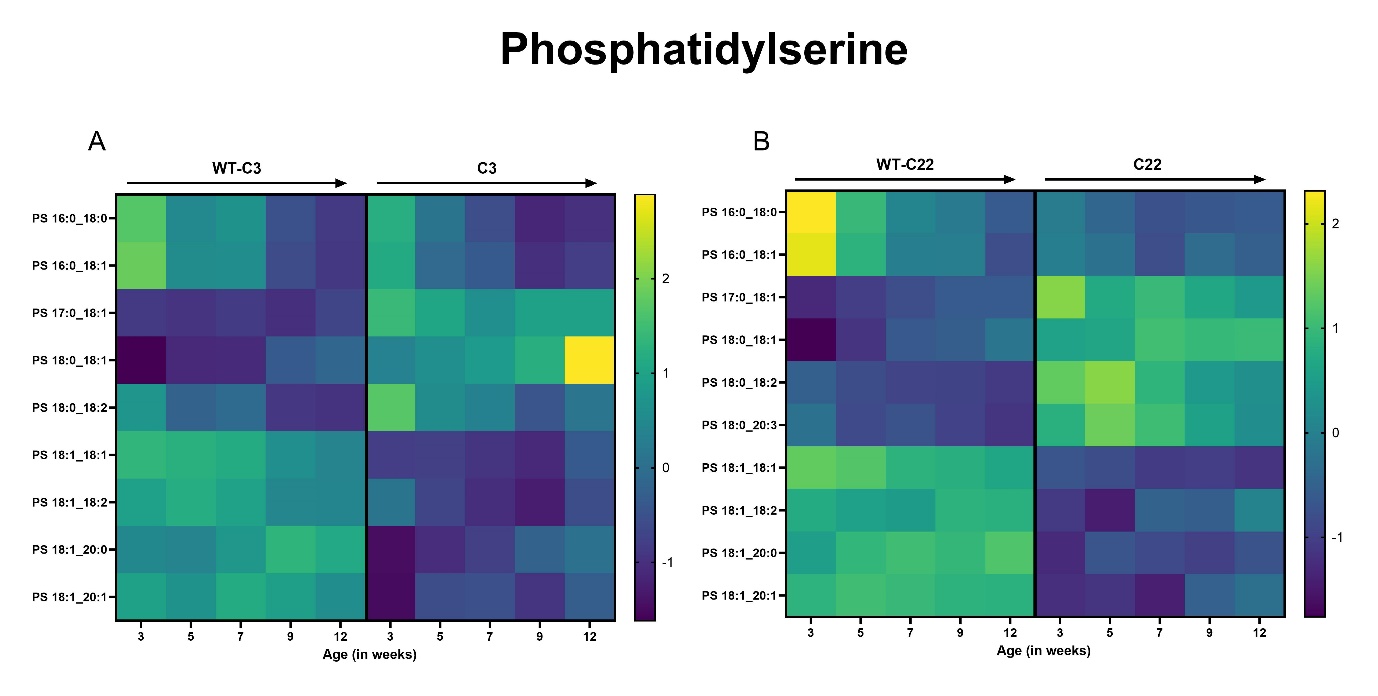


**Supplementary figure 11. Heatmap of z-scores for relative abundance of lipid species of phosphatidylserine (PS), measured in the sciatic nerve of 2 different CMT1A mouse models and controls over time.** The heatmap depicts z-scores of the lipid species present in each lipid class, normalized for the total concentration of that lipid class. Lipid species of which the average, both the CMT1A model and WT combined, were below 1% of the concentration of the lipid class are not depicted in this heatmap. Showing shifts in the lipid species present in PS for the WT-C3, C3, WT-C22 and the C22 at 2, 5, 7, 9 and 12 weeks of age. The data shows a shift from oleic acid (18:1) towards stearic acid (18:0) for both the C3 (A) and the C22 (B) compared to the corresponding wildtypes.

**
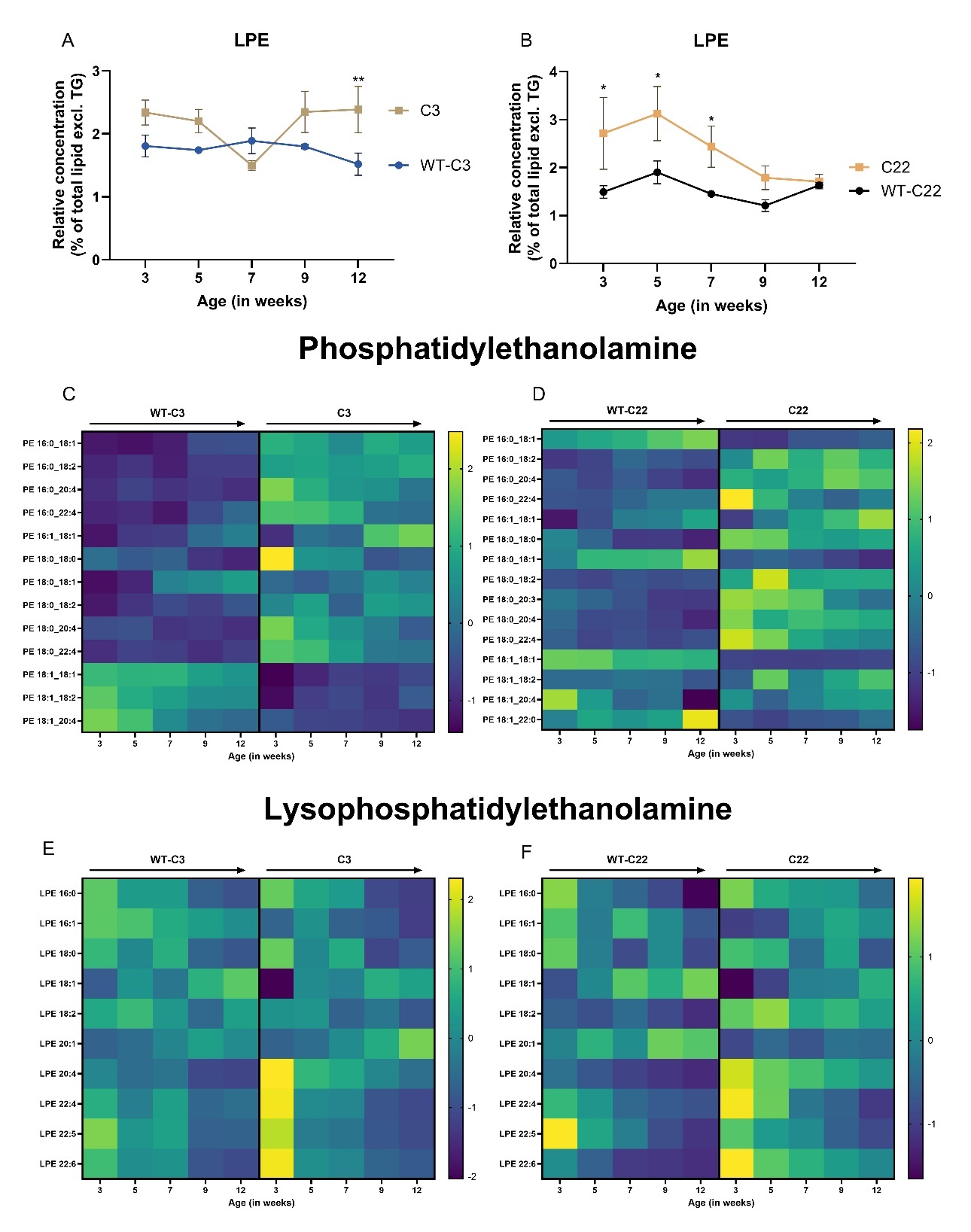
**
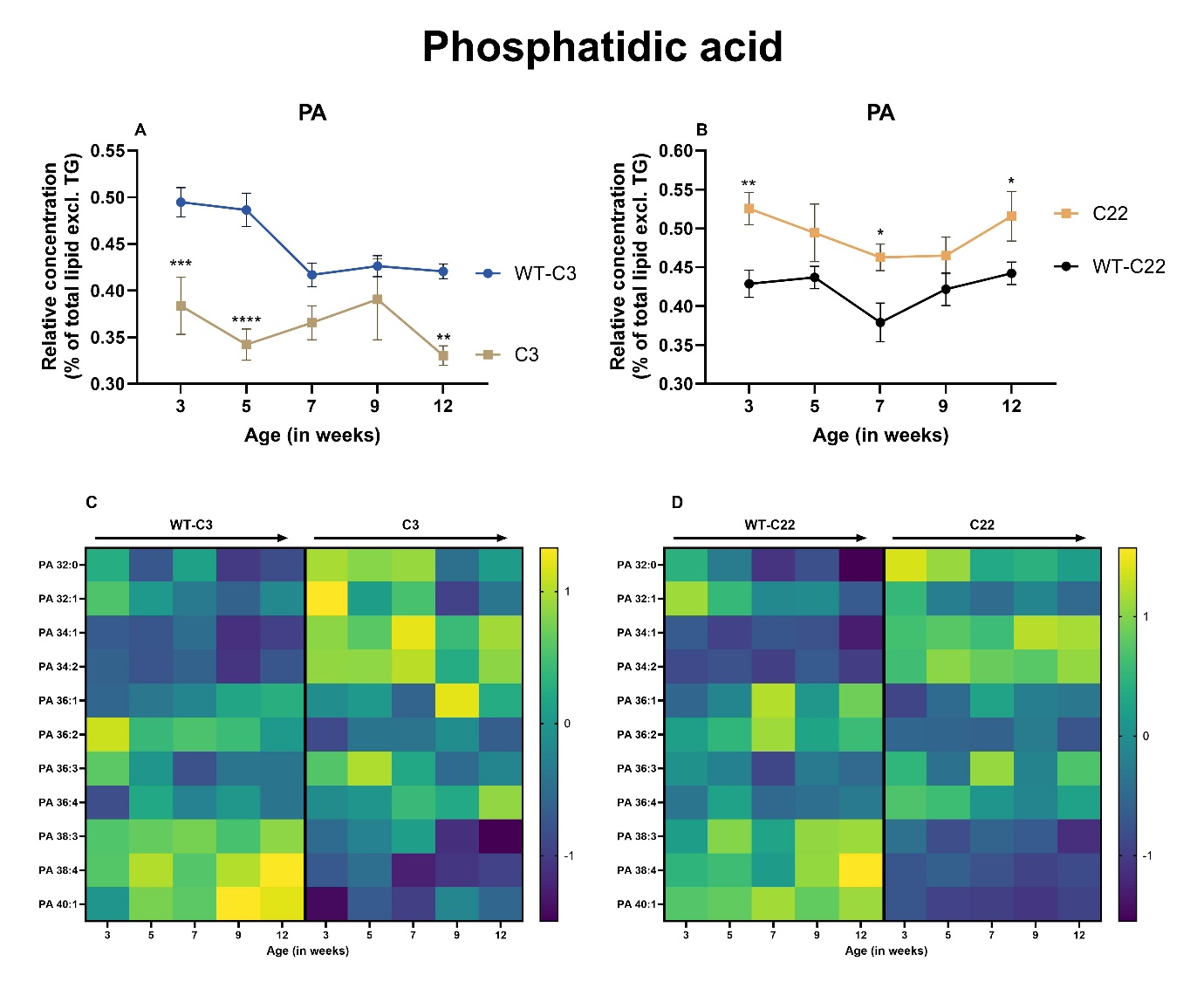


**Supplementary figure 12. Relative concentration of lysophosphatidylethanolamine (LPE) and heatmaps of z-scores for relative abundance of phosphatidylethanolamine (PE) and LPE, measured in the sciatic nerve of 2 different CMT1A mouse models and controls over time.** A & B: LPE concentration per mg protein divided by the lipidome (excluding TG) over time in the sciatic nerve of the C3 (A) and C22 (B) and the corresponding controls. Data is visualized as the mean ± SEM, analysis was preformed using a two-way ANOVA with a Tuckey post hoc analysis (*P<0.05, **P < 0.002). C-F: The heatmap depicts z-scores of the lipid species present in each lipid class, normalized for the total concentration of that lipid class. Lipid species of which the average, both the CMT1A model and WT combined, were below 1% of the concentration of the lipid class are not depicted in this heatmap. Showing shifts in the lipid species present in PE and LPE for the WT-C3, C3, WT-C22 and the C22 at 2, 5, 7, 9 and 12 weeks of age. The data shows a shift from mono-unsaturated fatty acids towards ploy-unsaturated and saturated fatty acids for both the C3 and the C22 compared to the corresponding wildtypes.

**Supplementary figure 13. Relative concentration of phosphatidic acid measured in the sciatic nerve of 2 different CMT1A mouse models.** A & B: Relative concentration of phosphatidic acid (PA) in the sciatic nerve of the C3 and C22 and corresponding controls**.** Data is visualized as the mean ± SEM, analysis was preformed using a two-way ANOVA with a Tuckey post hoc analysis (*P<0.05, **P < 0.002, ***P<0.0002, **** P<0.0001). C & D: The heatmap depicts z-scores of the lipid species present in each lipid class, normalized for the total concentration of that lipid class. Lipid species of which the average, both the CMT1A model and WT combined, were below 1% of the concentration of the lipid class are not depicted in this heatmap. Showing shifts in the lipid species present in PA for the WT-C3, C3, WT-C22 and the C22 at 2, 5, 7, 9 and 12 weeks of age. The data shows a shift to shorter fatty acids for both the C3 and the C22 compared to the corresponding wildtypes.


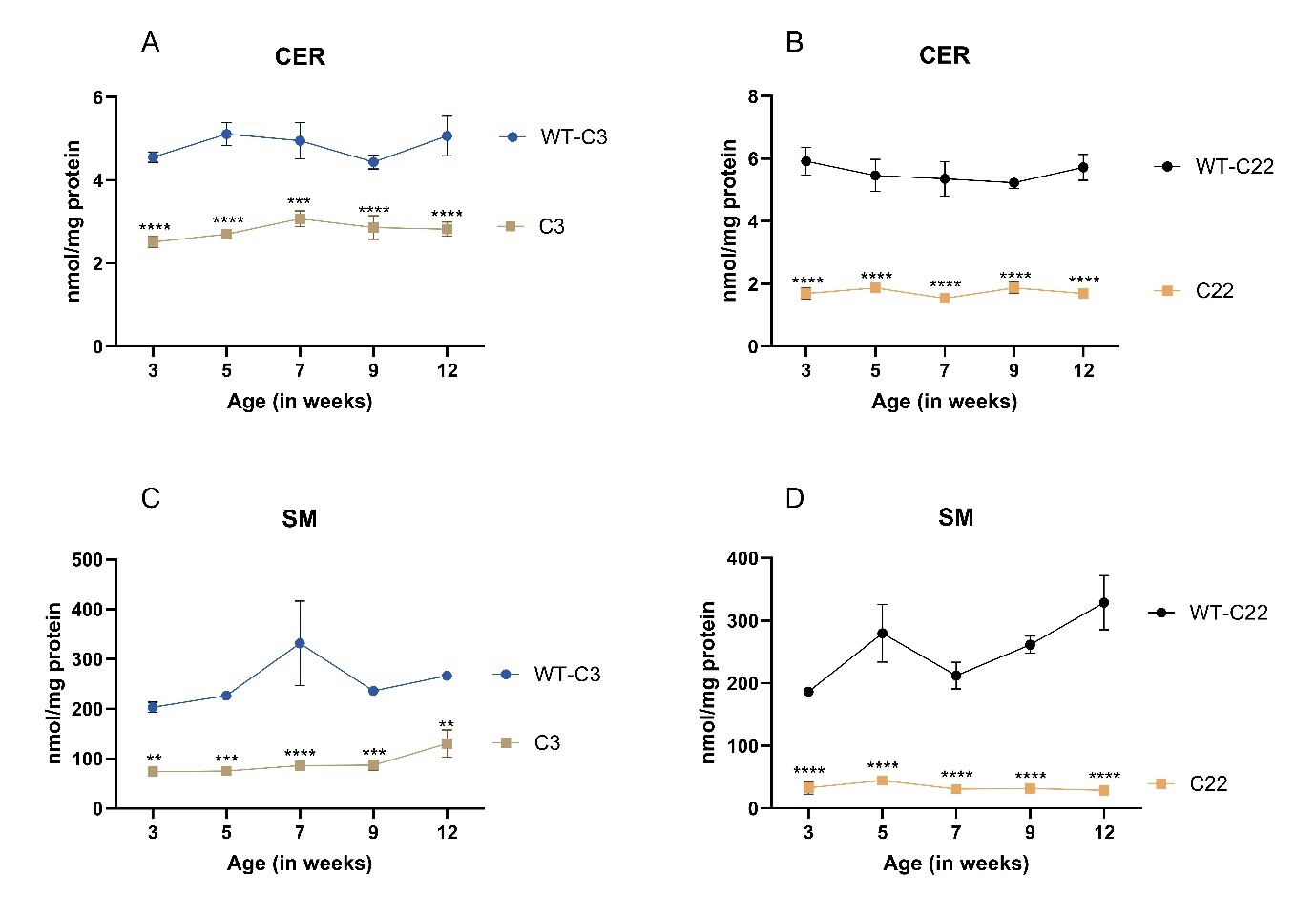


**Supplementary figure 14. Ceramide and sphingomyelin concentration over time in the sciatic nerve of CMT1A mouse models.** The Ceramide (CER) concentration per mg protein of the C3 (A) and C22 (B) mouse models and the corresponding controls at the 5 time points. C & D: The Sphingomyelin (SM) concentration per mg protein is consistently lower in all the C3 and C22 mice models compared to the controls. Data is visualized as the mean ± SEM, analysis was preformed using a two-way ANOVA with a Tuckey post hoc analysis (**P < 0.002, ***P<0.0002, ****P<0.0001).


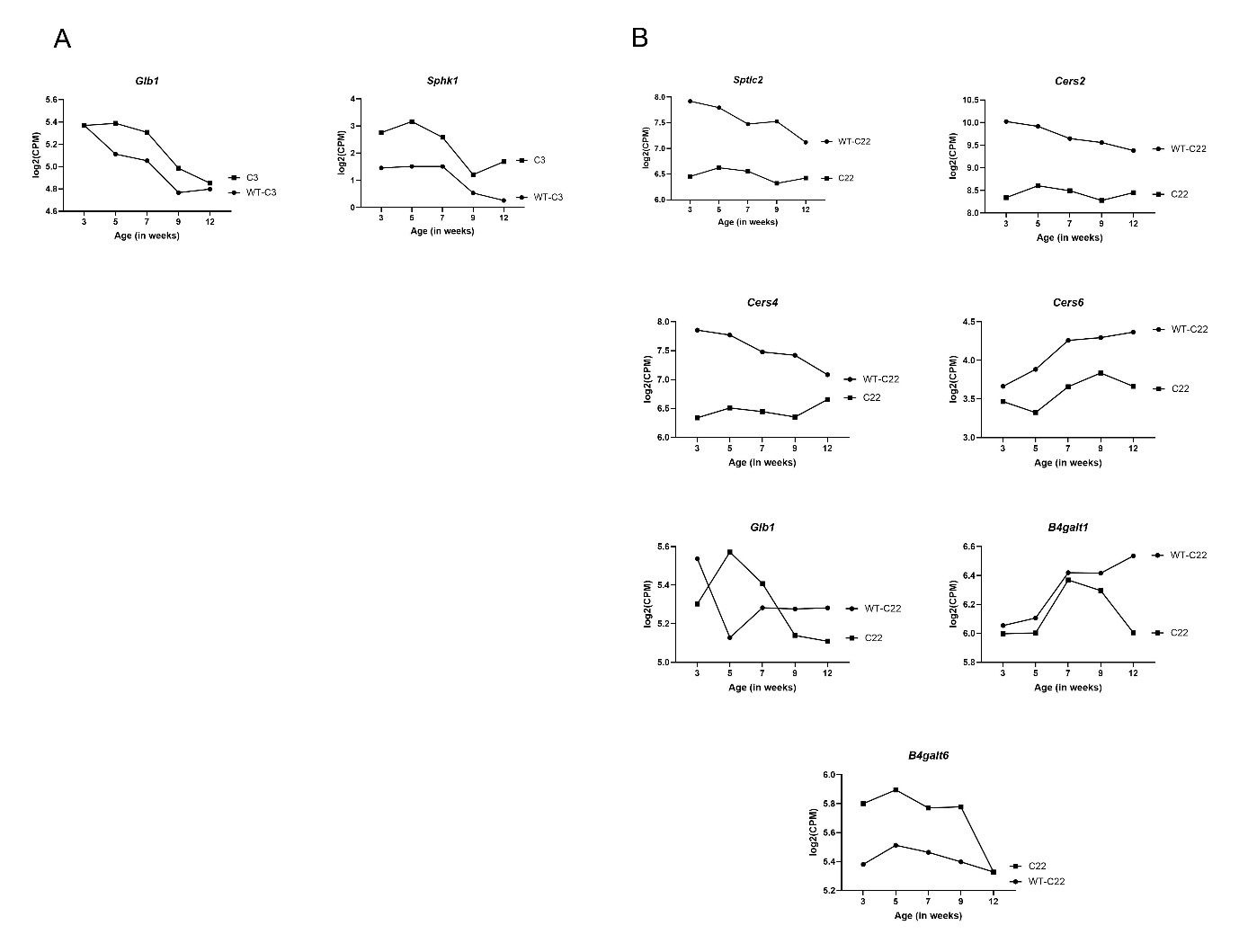


**Supplementary figure 15. Time course analysis of genes important for de novo sphingomyelin and ceramide synthesis.** Visualized are the expression levels of genes that play a pivotal role in Sphingomyelin and Ceramide synthesis over time for C3 (A) and C22 (B) and the corresponding wildtypes. The expression of genes depicted are effected by time in one of the groups. *Cers2, Ceramide Synthase 2; Cers4, Ceramide Synthase 4; Cers6, Ceramide Synthase 6; Sptlc2, Serine Palmitoyltransferase Long Chain Base Subunit 2; B4galt1, Beta-1,4-Galactosyltransferase 1; B4galt6, Beta-1,4-Galactosyltransferase 6 ; Glb1, Galactosidase Beta 1; Sphk1, Sphingosine kinase 1.*


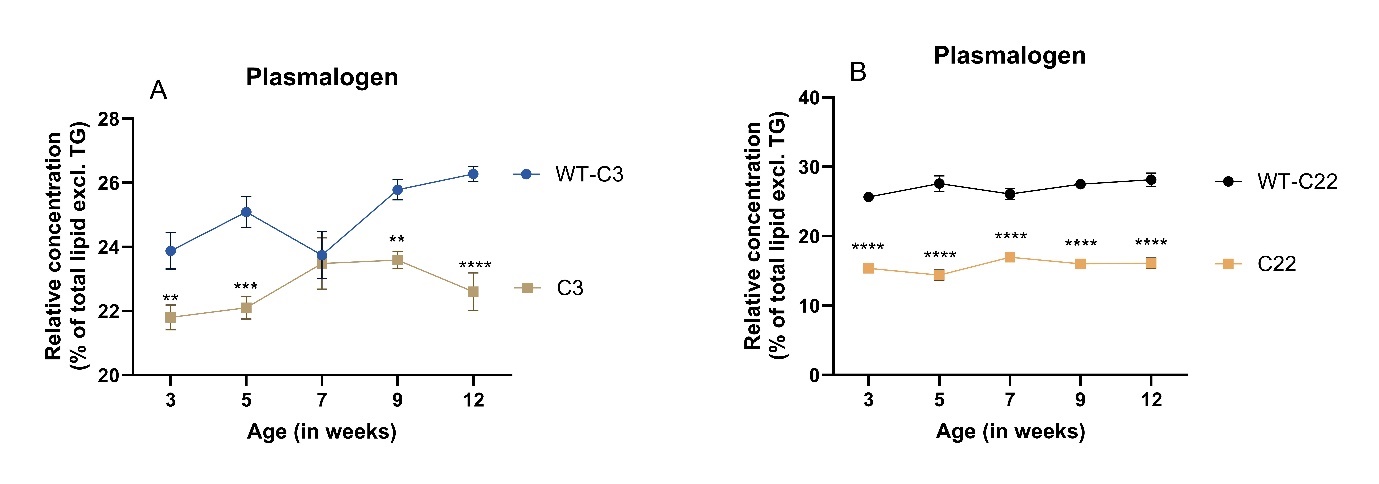


**Supplementary figure 16. Proportion of plasmalogen concentration of total lipid without triglyceride concentration of the sciatic nerve of CMT1A mouse models and the corresponding control over time without.** The plasmalogen concentration is consistently higher in WT-C22 than the C22 (B), while this is not the case for the C3 compared to the WT-C3 (A). The proportional concentration of C3 is lower than in the control at all ages except 7 weeks of age. Data is visualized as the mean ± SEM, analysis was preformed using a two-way ANOVA with a Tuckey post hoc analysis (*P<0.05, **P < 0.002, ***P<0.0002, ****P<0.0001).
